# Comprehensive characterization of pathogenic missense CTRP6 variants and their association with cancer

**DOI:** 10.1186/s12885-025-13685-0

**Published:** 2025-02-20

**Authors:** Muhammad Zubair Mehboob, Arslan Hamid, Jeevotham Senthil Kumar, Xia Lei

**Affiliations:** 1 Department of Biochemistry and Molecular Biology, Oklahoma State University, Stillwater, OK 74078 USA; 2 Institute for Molecular Biomedicine, Department of Molecular Immunology and Cell Biology, University of Bonn, 53115 Bonn, Germany; 3 142F Noble Research Center, Oklahoma State University, Stillwater, OK 74078 USA

**Keywords:** CTRP6, C1q domain, Missense variants, Pathogenicity, Cancer

## Abstract

**Background:**

Previous genome-wide association studies have linked three missense single nucleotide polymorphisms (SNPs) in C1q/TNF-related protein 6 (CTRP6) to diseases such as type 1 diabetes and autoimmune diseases. However, the potential association of newly identified missense CTRP6 variants with diseases, especially cancer, remains unclear.

**Methods:**

We used several pathogenicity prediction algorithms to identify deleterious mutations within the highly conserved C1q domain of human CTRP6, following the retrieval of all SNPs from the Ensembl database. We systematically analyzed the effects of these mutations on the protein’s stability, flexibility, structural conformation, compactness, stiffness, and overall functionality using various bioinformatics tools. Additionally, we investigated the association of these mutations with different cancer types using the cBioPortal and canSAR databases.

**Results:**

We identified 11 detrimental missense SNPs within the C1q domain, a region critical for this protein’s functionality. Using various computational methods, we predicted the functional impact of these missense variants and assessed their effects on the stability and flexibility of the CTRP6 structure. Molecular dynamics simulations revealed significant structural differences between the native and mutated structures, including changes in structural conformation, compactness, solvent accessibility, and flexibility. Additionally, our study shows a strong association between two mutations, G181S and R247W, and certain types of cancer: colon adenocarcinoma and uterine corpus endometrial carcinoma, respectively. We also found that the mutational status of CTRP6 and other cancer-related genes, such as MAP2K3, p16, TP53, and JAK1, affected each other’s expression, potentially contributing to cancer development.

**Conclusions:**

Our screening and predictive analysis of pathogenic missense variants in CTRP6 advance the understanding of the functional implications of these mutations, potentially facilitating more focused and efficient research in the future.

**Supplementary Information:**

The online version contains supplementary material available at 10.1186/s12885-025-13685-0.

## Background

In recent decades, the overall cancer mortality rate has decreased, reflecting significant advancements in medicine and healthcare. Despite these positive strides, cancer continues to pose a substantial global health burden and remains one of the leading causes of death worldwide. Regardless of cancer subtype, genetic mutations are a fundamental factor in the development of cancer, unifying various types and underscoring the imperative for comprehensive genetic research [1].

C1q/TNF-related proteins (CTRPs), a highly conserved family comprising 15 secreted proteins, have gained considerable attention in recent years. Despite structural and sequence similarities to adiponectin, an insulin-sensitizing hormone, each CTRP family member has a distinct tissue expression profile and plays a unique role in regulating physiological and pathological processes, including glucose and lipid metabolism [2–10]. Notably, CTRPs play a pivotal role in the development and progression of various cancer types, such as liver, colon, and lung cancers [11]. This connection is attributed to the independent contributions of diabetes, obesity, and insulin resistance to increased tumor risk. Several CTRPs, including CTRP1 [12, 13], CTRP3 [14], CTRP6 [15, 16], and CTRP8 [17], have been identified as contributors to tumor advancement through the activation of various signaling pathways. Therefore, CTRPs hold promise as both diagnostic markers and therapeutic targets for certain cancers.

CTRP6 is widely expressed in various human tissues, including the placenta, uterus, liver, fat, lung, and skin [7, 18]. It features four distinct domains: an N-terminal signal peptide, a short variable region, a collagen domain, and a C-terminal globular domain that is homologous to the complement protein C1q. Previous studies have revealed the involvement of CTRP6 in various physiological processes such as lipid metabolism [19], glucose regulation [7], cardiometabolism [20], inflammatory responses [21], and autoimmunity [22]. However, its role in cancer remains an emerging area of research. As early as 2011, CTRP6 was shown to be prominently localized in hepatocarcinoma cells but absent in normal liver tissues of human patients [23]. Subsequent in vitro experiments demonstrated that siRNA-mediated inhibition of CTRP6 impedes cell survival, migration, and invasion while promoting apoptosis by inactivating the AKT signaling pathway in hepatocellular carcinoma cells [24]. More recently, CTRP6 was found to be overexpressed in gastric carcinoma, contributing to cancer cell proliferation and migration [25]. Additionally, CTRP6 has been identified as a novel biomarker for predicting survival in patients with lung adenocarcinoma [26–31]. It has also been recognized as a diagnostic biomarker for clear cell renal cell carcinoma [32] and a predictor of poor prognosis in bladder cancer [33]. Thus, mutations in CTRP6, particularly missense mutations, may affect various functions and processes, potentially influencing protein-protein interactions with its yet unidentified receptor.

Identifying new variants in target genes typically relies on gene sequencing. However, the increasing number of newly discovered missense variants makes assessing their pathogenicity more challenging. This complexity arises because not all mutations impact protein function, and those that do can have varying effects on disease progression. Bioinformatics prediction methods often use alignments of homologous and distantly related sequences to assess amino acid conservation. Positions conserved throughout evolution are likely to have a significant impact on protein structure and function. To date, over 10 variants in CTRP6 have been associated with conditions such as type 1 diabetes and Graves’ disease through genome-wide association studies (GWAS). These include three missense SNPs: rs229527 (G21A/V; in the signal peptide) [34–37], rs229526 (P42R/H; in the signal peptide) [38], and rs7290488 (G55D; in the variable region) [39]. Notably, none of these variants have been identified within the C1q domain of CTRP6, which is considered a functional domain. In this study, we identified the most damaging variants of CTRP6 within its C1q domain by accessing the Ensembl variant database and utilizing various computational tools. These tools assess distinct features of protein residues to predict potential detrimental effects. Our primary goal is to pinpoint these harmful variants to better understand the functional implications of CTRP6 mutations in cancer development. This understanding may ultimately aid in efforts to restore protein function and mitigate the associated disease.

## Methods

### Dataset collection and processing

A complete list of human CTRP6 variants was retrieved from the Ensembl database [40] and categorized into two groups: non-coding variants (regulatory SNPs mapped to non-coding regions such as 3’ and 5’ UTRs, splice sites, and introns) and coding variants (synonymous, missense, and nonsense SNPs). The number of coding variants is significantly smaller compared to non-coding variants (1173 vs. 25,843) (Fig. S1). Among the coding variants, nearly half are missense variants. After selecting the canonical transcript of CTRP6 and removing duplicates, 233 missense SNPs were further analyzed to predict their pathogenicity using various bioinformatics tools with different backend algorithms. Additionally, the potentially deleterious SNPs were mapped onto the protein domains of CTRP6 to gain insights into their potential functional consequences.

### Pathogenicity prediction

To assess the pathogenicity of missense SNPs, we used seven algorithm-based online tools, which require protein sequence and SNP information as input. These tools include PolyPhen-2 [41], SIFT [42], PhD-SNP^g^ [43], SNAP2 [44], MutPred2 [45], SNPs&GO [46], and Meta-SNP [47]. These predictive tools categorize variant pathogenicity as either “neutral” or “damaging” and assign scores based on tool-specific scales. The sensitivity, specificity, and accuracy of these tools have been evaluated in prior studies [48, 49], and the detailed methods for their application are described in previous publications [50–52]. Briefly, PolyPhen-2 combines sequence-based features, structural information and multiple sequence alignments (MSA) and employs a Naïve Bayes classifier to estimate the probability of a mutation being damaging. SIFT predicts the potential pathogenicity based on conservation score derived from MSA. PhD-SNP^g^ uses a Gradient Boosting-based algorithm (trained on ~ 104,000 Clinvar-derived variants) together with sequence context and evolutionary conservation scores to predict detrimental effect of single nucleotide variant. A neural network-based tool, SNAP2, predicts the functional impact of substituted amino acids by leveraging protein sequence and structural features, achieving high accuracy (83%) in distinguishing between neutral and damaging variants. MutPred2 integrates sequence-based features, evolutionary conservation, and structural/functional properties of proteins. It is trained using 53,180 pathogenic and 206,946 neutral variants. SNPs&GO is based on support vector machine classifier analyzing features such as protein sequences information, evolutionary data and Gene Ontology-based functional annotations to prioritize disease-related variants. Meta-SNPs, a random forest-based algorithm, takes output of the four tools (PANTHER, PhD-SNP, SIFT and SNAP) as an input and provides a consensus prediction to discriminate detrimental variants.

### Evolutionary conservation analysis 

To highlight the evolutionary significance of the wild-type amino acids, we performed a conservation analysis using ConSurf [53]. This tool uses the position-specific iterative Basic Local Alignment Search Tool (PSI-BLAST) to identify and align closely related sequences and calculates conservation scores using a Bayesian approach. The conservation score is measured on a nine-point scale, with higher scores indicating greater conservation and lower scores reflecting increased variability at a given position. Specifically, scores of 1–3 denote variable regions, 4–6 represent average conservation, and 7–9 indicate highly conserved residues.

### Structure modeling and validation

The 3D structure of CTRP6 was obtained from the AlphaFold database, an AI-based system that uses a deep neural network approach [54]. The confidence of the protein models is represented by the pLDDT (predicted Local Distance Difference Test) score. The CTRP6 C1q domain (amino acids 144–275) demonstrates a high confidence score (pLDDT > 90). Additionally, the region 113–143 exhibits a medium confidence score (70 < pLDDT < 90), while the region 1-112 shows low to very low confidence scores (pLDDT < 70). AlphaFold’s predicted aligned error (PAE) plot illustrates the expected distance errors in Ångströms, aiding in predicting the relative positions of domains within proteins. The CTRP6 model was validated using a Ramachandran plot generated on the MolProbity server and further refined through energy minimization via the “steepest descent” approach in GROMACS [55]. After introducing mutations into CTRP6, the Phyre2 server was used to model the mutated structures. Subsequently, the wild-type and mutated CTRP6 structures were superimposed in Discovery Studio to detect structural deviations and altered regions.

### Protein stability and flexibility prediction

To assess the impact of deleterious SNPs on CTRP6 protein stability, six methods were used to calculate changes in Gibbs free energy: I-Mutant2.0, MUpro, DUET, Impact of Non-synonymous mutations on Protein Stability - Multi Dimension (INPS-MD), Site Directed Mutator (SDM), and mutation Cutoff Scanning Matrix (mCSM). I-Mutant2.0 uses support vector regression to calculate the change in free energy (ΔΔG) caused by single-point mutations in a protein sequence. It considers various features, including solvent accessibility, sequence conservation, and secondary structure information [56]. MUpro uses a support vector machine (SVM) algorithm, trained on experimental data, to classify mutations as either stabilizing or destabilizing [57]. DUET combines two methods, SVM and mCSM, to assess the impact of SNPs on protein stability [58]. INPS-MD integrates molecular dynamics simulations with nonlinear spectral analysis, leveraging key characteristics of a protein’s three-dimensional structure to predict the impact of mutations on stability [59]. SDM employs a thermodynamic cycle-based approach to estimate the effect of single-site mutations. It quantifies mutation-associated free energy changes and provides a stability score, indicating whether a mutation stabilizes or destabilizes the protein [60]. mCSM calculates the difference in free energy associated with mutations using a computational approach based on graph theory and machine learning algorithms [58]. DynaMut assesses the impact of a mutation on both protein stability and dynamics by measuring vibrational entropy changes and capturing protein motions based on graph signatures [61]. Furthermore, based on vibrational entropy changes, DynaMut provides a visual representation of protein flexibility, using red hues to indicate areas of higher flexibility and blue hues to denote regions of greater rigidity.

### Molecular dynamics simulation

GROMACS was used to simulate the cellular environment and examine time-dependent structural changes in both wild-type and mutated models during protein transformation [55]. The initial models of proteins were prepared by pdb2gmx program in the GROMACS package, and simulation system was built by embedding protein structure in a dodecahedron-shaped water box, with boundaries at least 1.2 nm away from the solute surface to minimize boundary effects. Using TIP3P water model, solvation step was realized which is computationally efficient and reliable for reproducing bulk water properties [62]. Sodium (Na⁺) and chloride (Cl⁻) ions were added to neutralize the system and achieve a physiological ionic strength of 0.15 M. The system was run for 200 picoseconds using the steepest descent method to ensure stability, reaching an energy threshold of 1000KJ/mol/nm, followed by applying the CHARMM36 force field in GROMACS [63]. The system was equilibrated for 100 ps, keeping the volume and temperature (300 K) constant. Temperature coupling is done via velocity-rescaling thermostat with the coupling constant to 0.1 ps [64]. Molecular dynamics simulations were conducted for 100 nanoseconds (ns), starting at a temperature of 300 K with a random seed of -1. A long-range Van der Waals cut-off (rvdw) was set during the energy minimization stage, as described by Huang et al. [65]. GROMACS built-in options were used to calculate various structural parameters, including root-mean-square deviation (RMSD), root-mean-square fluctuation (RMSF), radius of gyration (Rg), solvent-accessible surface area (SASA), and hydrogen bonds (both intra- and inter-molecular). Visualizations regarding structural snapshots and trajectory were performed via VMD-Visual Molecular Dynamics [66], and further plotting was done using QtGrace software.

### Additional data assessment and analysis

In addition to structural assessments, various analyses were performed to elucidate the effects of deleterious variants on structural influence, disordered regions, physicochemical properties, and Gene Ontology (GO) terms. Point mutations can significantly impact the secondary structure of proteins by disrupting local amino acid interactions, which can ultimately affect the overall 3D conformation. To predict changes in secondary structures caused by these mutations, we used PSIPRED 4.0 [67]. Additionally, we employed the DISOPRED3 algorithm, which uses a neural network and nearest neighbor classifier, analyzing data from the Disprot and PDB databases to identify intrinsically disordered regions in proteins [68]. This algorithm assigns a confidence score to protein residues ranging from 0 to 1, with a cut-off value greater than 0.5 indicating disordered status. The grand average of hydropathy (GRAVY), indicating the overall hydrophobicity/hydrophilicity of a protein, was measured using ProtParam [69]. For GO terms analysis, GO terms were evaluated using the GO Resource [70], and heatmaps were generated with the standalone program TB-Tools [71].

### Correlation of missense SNPs with cancer

To investigate the correlation between the 11 deleterious missense SNPs and various cancer types, we accessed cancer genomics data through cBioPortal [72–74] and canSAR [75], both of which leverage data from The Cancer Genome Atlas (TCGA). The TNMplot database provides differential gene expression analysis across normal, tumor, and metastatic tissues by integrating data from GEO, GTEx, TCGA, and TARGET [76]. For the comparative analysis of CTRP6 gene expression, TNMplot RNA-seq data was chosen to compare samples from non-cancerous healthy individuals to those from cancer patients. The muTarget database serves as a valuable resource for establishing the connection between mutations and gene expression changes in solid tumors [77]. It provides two analysis options: ‘genotype’ analysis, which elucidates how mutations in the query gene (CTRP6) affect the expression of other genes in various cancer types, and ‘target’ analysis, which delineates how mutations in other genes lead to changes in the expression of the query gene. Both analyses were performed by selecting the coding mutation type.

## Results

### Pathogenicity prediction of CTRP6 missense SNPs

**Fig. 1:**
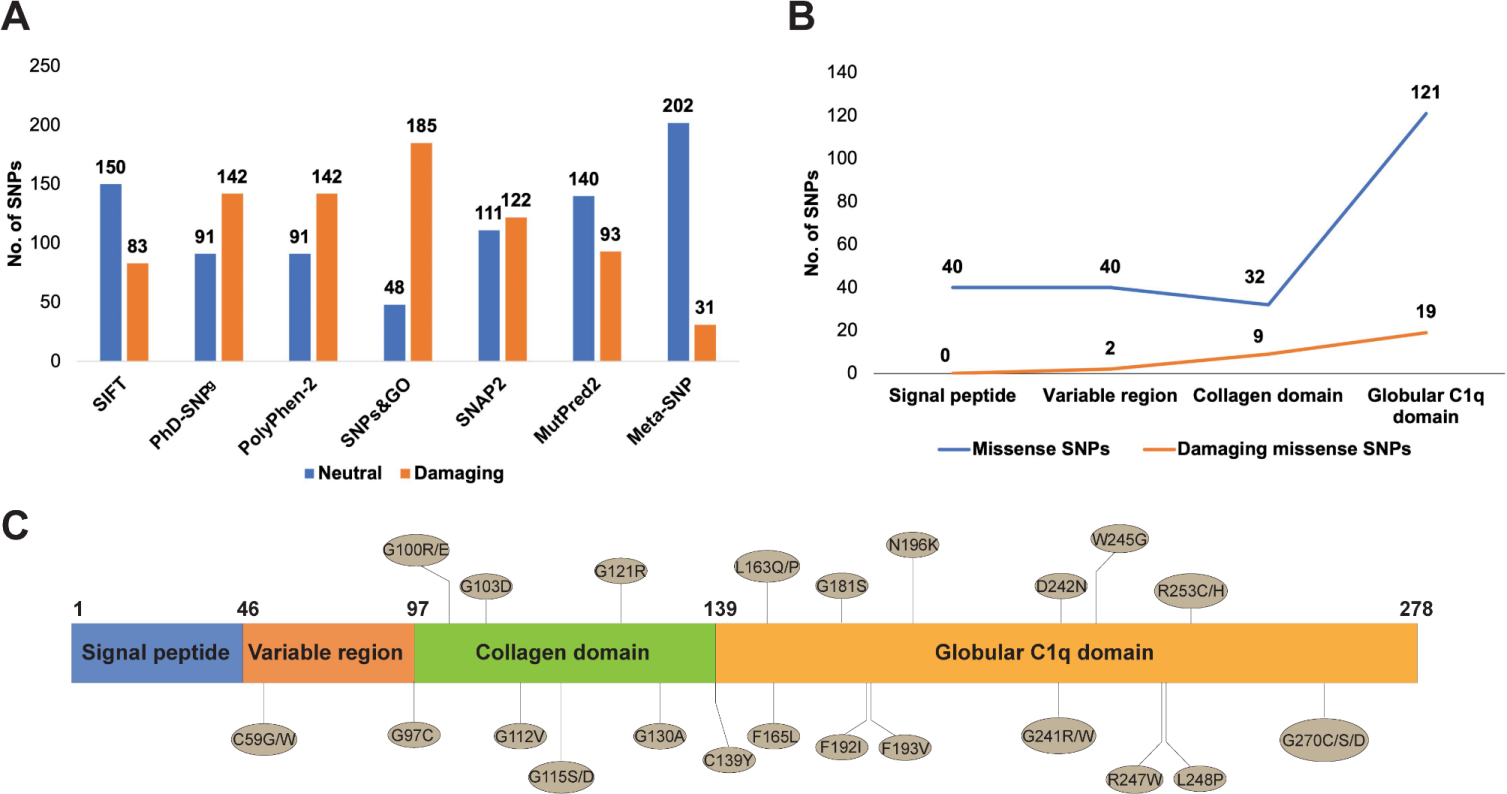
Deleterious missense SNPs identification and their domain mapping. **A**) Number of neutral and damaging missense SNPs predicted by seven tools with different algorithms. **B)** Number of damaging and total missense SNPs in each domain of human CTRP6 protein. These damaging missense SNPs were predicted by all of the seven tools. **C**) Mapping of all damaging SNPs to different domains of human CTRP6 protein.

All variants of human CTRP6 were retrieved from the Ensembl database (**Fig.**S1) and 233 missense SNPs were obtained. These missense SNPs were then screened using seven pathogenicity predictor tools trained on experimental data and statistical models. The results from these computational tools are summarized in Table S1 and S2. Each tool employs distinct statistical models for pathogenicity prediction, leading to varying percentages of neutral and damaging SNPs (Fig. 1A). We further mapped all the missense SNPs, especially those predicted as damaging, to the protein domains of CTRP6 (Fig. 1B, C). In the globular C1q domain, 19 of the 121 missense SNPs were identified as pathogenic by all seven prediction tools (Table 1). Notably, no pathogenic mutations were observed in the N-terminal signal peptide region. However, several pathogenic mutations were identified within the variable region and collagen domain. Given the crucial role of the C-terminal C1q domain in CTRP6 function, evidenced by observations such as the size of CTRP6 in mouse serum (~ 25 kDa) matching that of the isolated C1q head but not the full-length CTRP6 protein (38 kDa) [22, 78, 79], we selectively focused on the 19 detrimental SNPs within the C1q domain for further investigation.

**Table 1:** Pathogenicity of high-risk missense SNPs in the human CTRP6 C1q domain predicted by seven tools

SNP ID	AA change	SIFT	PhD-SNP^g^	PolyPhen-2	SNPs&GO	SNAP2	MutPred2	Meta-SNP
		score ≤ 0.05: Dam	score > 0.5: Dam	score 0.15 to 1.0: Dam	score > 0.5: Dis	score 0 to 100: Eff	score > 0.5: Dam	score > 0.5: Dis
rs781322736	C139Y	Dam	Dam	Dam	Dis	Eff	Dam	Dis
rs1398111940	L163Q	Dam	Dam	Dam	Dis	Eff	Dam	Dis
rs1398111940	L163P	Dam	Dam	Dam	Dis	Eff	Dam	Dis
rs145588561	F165L	Dam	Dam	Dam	Dis	Eff	Dam	Dis
rs140887252	G181S	Dam	Dam	Dam	Dis	Eff	Dam	Dis
rs765109174	F192I	Dam	Dam	Dam	Dis	Eff	Dam	Dis
rs145370378	F193V	Dam	Dam	Dam	Dis	Eff	Dam	Dis
rs200271327	N196K	Dam	Dam	Dam	Dis	Eff	Dam	Dis
rs754701333	G241R	Dam	Dam	Dam	Dis	Eff	Dam	Dis
rs754701333	G241W	Dam	Dam	Dam	Dis	Eff	Dam	Dis
rs149630379	D242N	Dam	Dam	Dam	Dis	Eff	Dam	Dis
rs762143456	W245G	Dam	Dam	Dam	Dis	Eff	Dam	Dis
rs774652920	R247W	Dam	Dam	Dam	Dis	Eff	Dam	Dis
rs763287603	L248P	Dam	Dam	Dam	Dis	Eff	Dam	Dis
rs201178136	R253C	Dam	Dam	Dam	Dis	Eff	Dam	Dis
rs1457358945	R253H	Dam	Dam	Dam	Dis	Eff	Dam	Dis
rs765588555	G270S	Dam	Dam	Dam	Dis	Eff	Dam	Dis
rs765588555	G270C	Dam	Dam	Dam	Dis	Eff	Dam	Dis
rs865840494	G270D	Dam	Dam	Dam	Dis	Eff	Dam	Dis

Dam: damaging; Dis: Disease; Eff: Effect

### Evolutionary conservation analysis and protein stability analysis

**Fig. 2:**
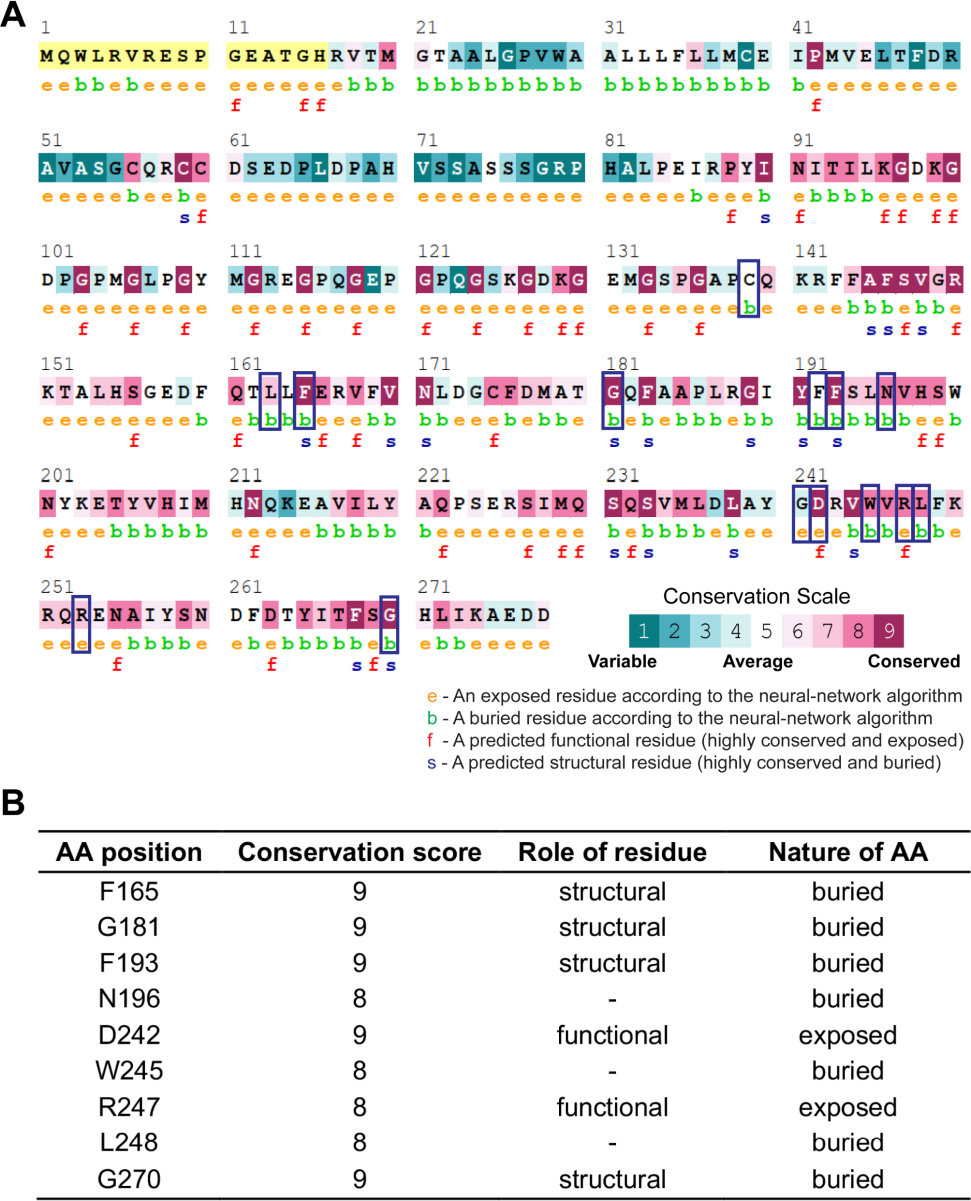
Prediction of evolutionary conserved amino acid residues in human CTRP6 by ConSurf server. **A)** Conservation scores are represented with color-coded bars. Deleterious missense SNPs in the C1q domain are marked by blue rectangles. **B**) Summary of the characteristics of amino acid residues where the deleterious SNPs are located.

Evolutionarily conserved residues are pivotal to protein stability, interaction interfaces, and functional sites. Hence, we investigated the conservation of native residues of human CTRP6 using the ConSurf server [53]. By leveraging MSA and protein structural data, ConSurf assesses the evolutionary pressure, solvent exposure, and possible functional and structural significance of each amino acid. Our analysis revealed that the globular C1q head is a more evolutionarily conserved domain, containing a higher number of conserved amino acids (Fig. 2A). Substitutions at highly conserved positions are more likely to disrupt protein structure and function than those at less conserved positions. Among the 14 wild-type positions with SNPs (19 missense SNPs, including 4 multiallelic sites), 9 residues exhibit high conservation across mammals, birds, and fishes (Fig. S2), with ConSurf conservation scores ranging from 8 to 9 (Fig. 2A). Therefore, 11 variants at these 9 highly conserved positions were selected for subsequent analysis. The higher degree of conservation at these positions supports the hypothesis that these substitutions could be detrimental, though this remains inconclusive. Notably, D242 and R247 are functionally exposed residues on the surface of the CTRP6 protein (Fig. 2B), suggesting their potential involvement in protein-protein interactions. In contrast, the remaining 7 residues are expected to be buried, likely contributing to the protein’s structural integrity.

To evaluate the functional impact of these 11 identified deleterious SNPs on CTRP6 stability, we employed six protein-stability predictive servers to assess the ΔΔG (change in Gibbs free energy) values. Notably, 9 out of the 11 SNPs were consistently predicted by all six servers to destabilize the C1q domain (Table 2). Consequently, we categorized the 11 SNPs into two groups: damaging (R247W and G270C) and most damaging missense SNPs (F165L, G181S, F193V, N196K, D242N, W245G, L248P, G270S, and G270D). The collective analysis from these servers suggests that these point mutations may induce conformational changes, potentially leading to alterations in protein stability.

**Table 2:** Effect of substituted amino acids on the stability of CTRP6 C1q domain predicted by six tools

AA change	I-Mutant2.0	ΔΔG	MUpro	ΔΔG	INSP-MD	ΔΔG	DUET	ΔΔG	SDM	ΔΔG	mCSM	ΔΔG
F165L	Dec	-2.97	Dec	-0.624	Dec	-2.295	Destab	-1.615	Destab	-2.54	Destab	-1.367
G181S	Dec	-1.45	Dec	-0.538	Dec	-0.753	Destab	-1.793	Destab	-4.13	Destab	-1.381
F193V	Dec	-2.53	Dec	-1.32	Dec	-2.116	Destab	-1.22	Destab	-1.65	Destab	-1.027
N196K	Dec	-1.44	Dec	-1.33	Dec	-0.317	Destab	-0.305	Destab	-1.7	Destab	-0.24
D242N	Dec	-2.43	Dec	-1.07	Dec	-0.513	Destab	-0.523	Destab	-0.32	Destab	-0.695
W245G	Dec	-2.94	Dec	-1.37	Dec	-2.512	Destab	-3.753	Destab	-2.14	Destab	-4.295
R247W	Dec	-0.59	Dec	-0.551	Dec	-0.807	Destab	-0.51	*Stab*	0.43	Destab	-0.642
L248P	Dec	-2.2	Dec	-2.325	Dec	-2.94	Destab	-2.943	Destab	-4.31	Destab	-2.26
G270S	Dec	-1.94	Dec	-1.322	Dec	-0.475	Destab	-0.575	Destab	-2.44	Destab	-0.46
G270C	Dec	-2.13	Dec	-1.17	Dec	-1.822	*Stab*	0.616	Destab	-0.05	*Stab*	0.394
G270D	Dec	-2.58	Dec	-1.064	Dec	-1.01	Destab	-1.254	Destab	-1.8	Destab	-1.209

Dec: Decrease; Destab: Destabilizing; Stab: Stabilizing

### Flexibility analysis of wild-type and mutant proteins

**Fig. 3:**
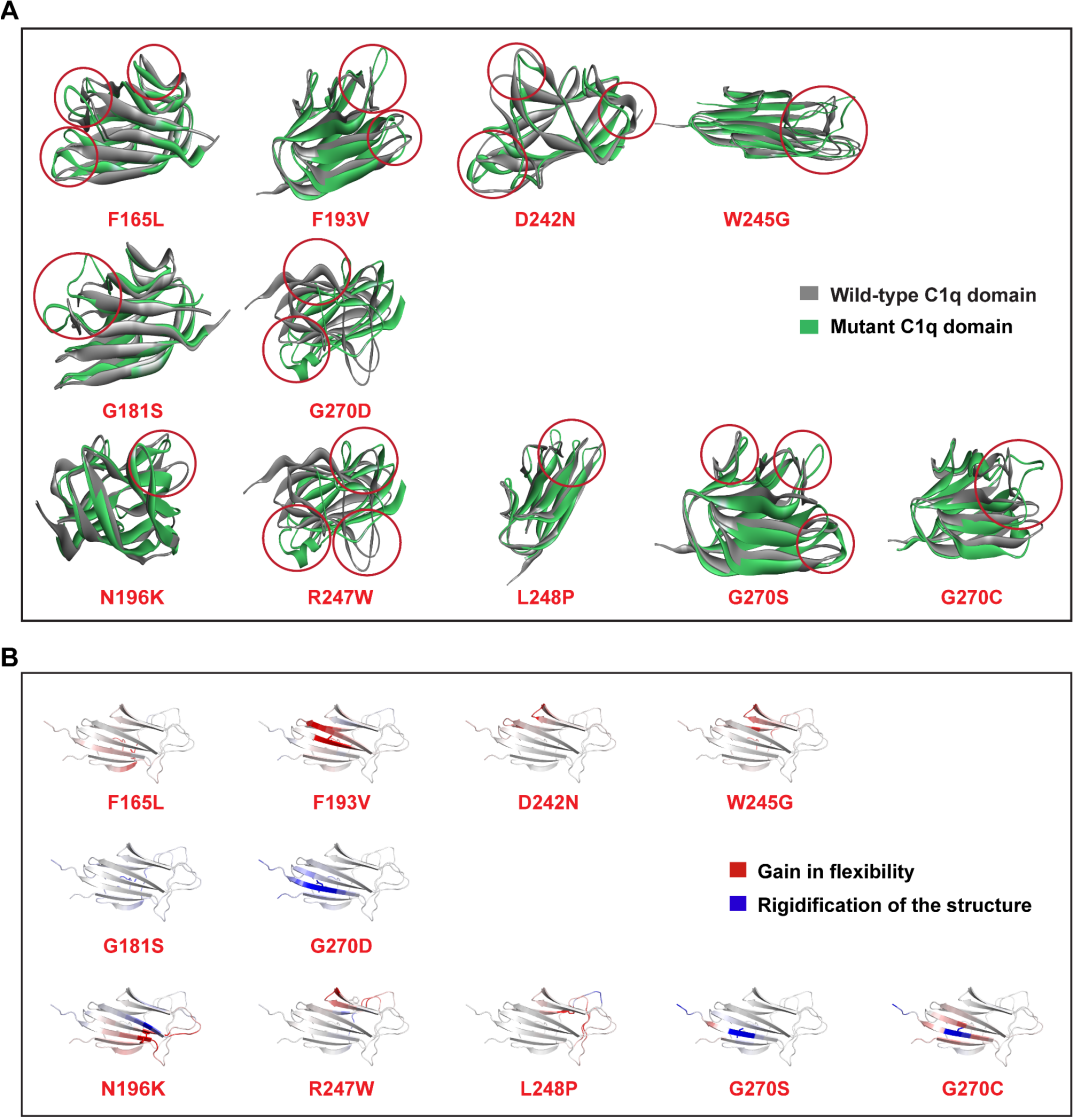
Comparative analysis of wild-type and mutated C1q structures to highlight structural variations and flexibility patterns. **(A)** Disordered regions are indicated by red circles in superimposed native and mutated C1q structures. Mutant proteins exhibit significant deviations in structural loops from the native structure. **(B)** Local alterations in flexibility, either increased (red) or decreased (blue), are influenced by the substituted amino acids in various areas.

To further assess the impact of damaging missense SNPs on protein structure, we isolated the C1q domain of human CTRP6 from the AlphaFold model, subjected it to an energy minimization step, and examined its structural quality using a Ramachandran plot. Surprisingly, all 140 residues of the domain were found in the allowed regions (> 99.8%) on the Ramachandran plot, suggesting that the C1q structure is well-folded and stable. Structural superimposition of the normal and 11 variant models revealed notable alterations in loops, secondary structures, and complete non-overlapping of some β-sheets within the C1q domain (Fig. 3A).

Furthermore, we examined the alterations in protein flexibility induced by SNPs using the DynaMut software, which estimates ENCoM (Elastic Network Contact Model) values. This analysis allowed us to assess the energetic contribution of individual residues to protein stability, providing insights into the impact of mutations on protein function. The changes in protein rigidity and flexibility across different regions of the mutated C1q domain of CTRP6 are depicted in Fig. 3B. We observed an increase in molecular flexibility, driven by elevated vibrational entropy, in four variants (F165L, F193V, D242N, W245G), whereas two substitutions (G181S, G270D) resulted in reduced molecular flexibility. Among the remaining five variants (N196K, R247W, L248P, G270S, G270C), some regions exhibited increased flexibility, while others experienced rigidification. This shift in flexibility has the potential to disrupt protein interactions and alter the conformation of the active site. In summary, damaging missense SNPs induce significant structural and flexibility changes in the C1q domain, likely disrupting protein interactions and impairing protein function.

### Molecular dynamics (MD) simulation analysis

**Fig. 4:**
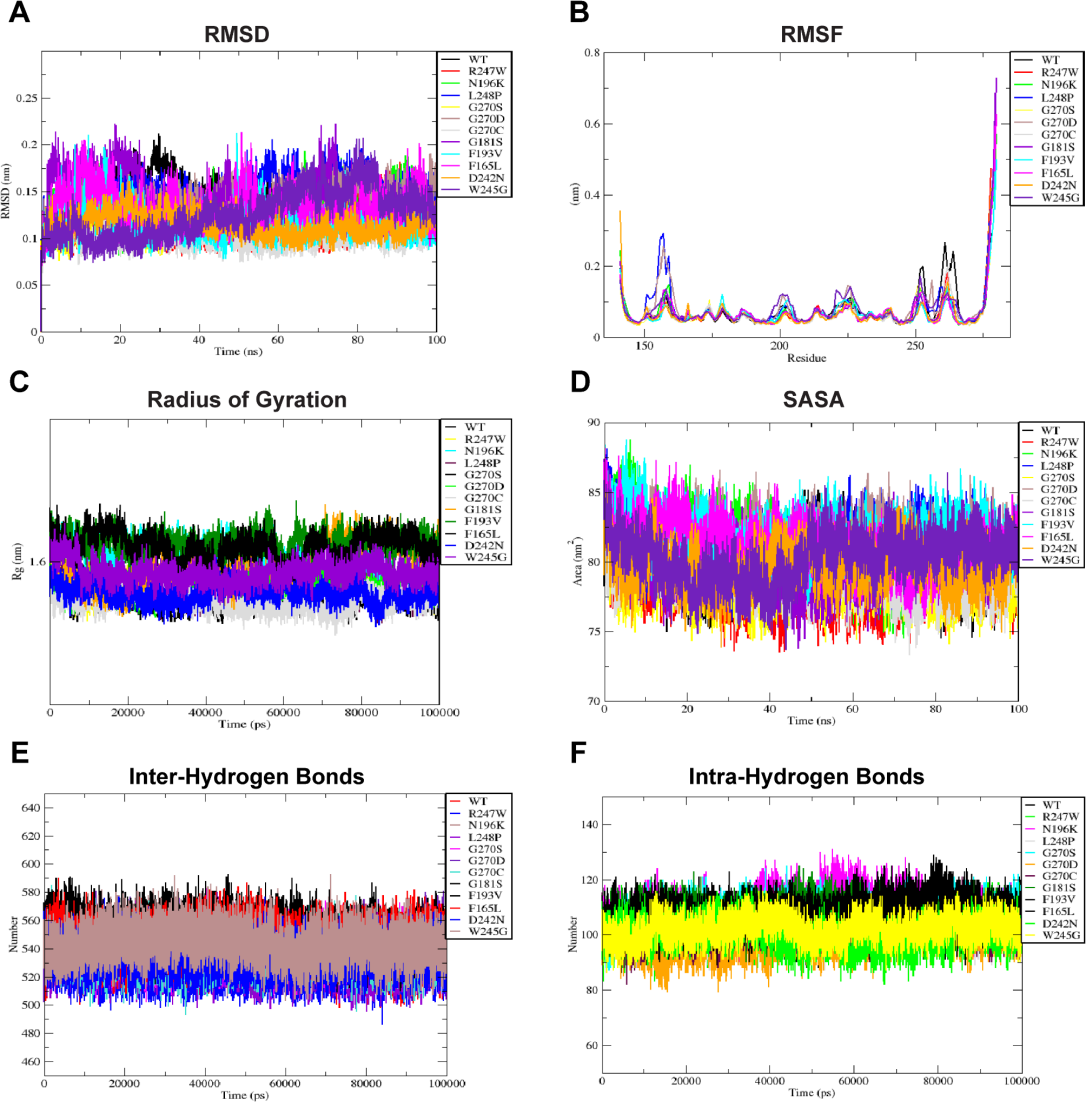
Molecular simulation results of wild-type and variant C1q domain of CTRP6 at 100 ns time periods. **A)** RMSD shows the overall structural deviation of Cα atoms in wild-type and mutant structures. **B**) RMSF indicates local dynamic changes, represented by a single Cα value over the entire stimulated structure. **(C)** Radius of gyration reflects the compactness of models. **(D)** SASA measures the total solvent-exposed surface. **E**, **F)** Formation of new and breakage of existing intermolecular and intramolecular hydrogen bonds.

**Fig. 5:**
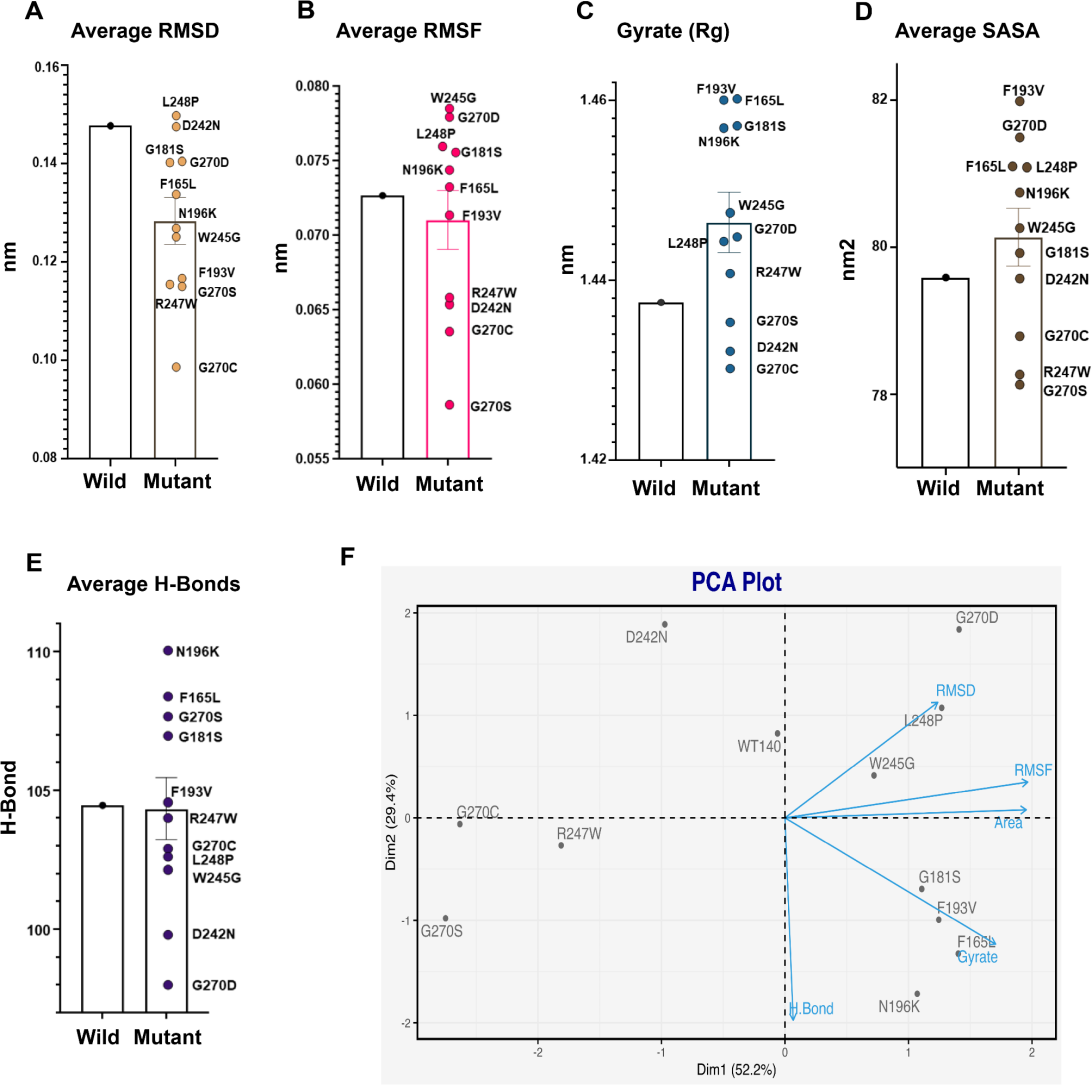
Scatter plots of average values of MD parameters of wild-type and mutated C1q structures and principal component analysis (PCA). **(A)** Average RMSD values. **(B)** Average RMSF values. **(C)** Average Rg values. **(D)** Average SASA values. **(E)** Average number of hydrogen bonds, including both intermolecular and intramolecular ones. **(F)** PCA plot allows visualizing conformational diversity.

To comprehensively explore the influence of variants on the conformation, compactness, solvent accessibility, flexibility, and stability of the C1q domain of CTRP6, we conducted extensive MD simulations spanning 100 ns. RMSD analysis was used to assess the deviation in the movement of native and mutant backbones within a physiological environment (Fig. 4A and Fig. S3), providing valuable insights into how the 11 deleterious missense SNPs alter the structural dynamics of the C1q domain. At around 40 ns into the simulation, the backbone Cα atoms of the wild-type C1q domain stabilized at approximately 0.15 nm, while the mutant models reached equilibrium by 20 ns, except for L248P and W245G. Although all mutant structures remained largely comparable to the native C1q structure, F165L, G181S, F193V, and N196K displayed instability, as reflected in their RMSD values ranging between ~ 0.15 and ~ 0.20 nm in the first 10 ns. The deviation of other variants from the wild-type RMSD also occurred at different timeframes, with the average RMSD presented in Fig. 5A. Furthermore, RMSF analysis revealed notable distinctions in fluctuation patterns between wild-type and variant structures, particularly evident at residues located at the start and end of the C1q domain (Fig. 4B and Fig. S4). The mutants L248P and G270D exhibited a comparatively flexible region spanning residues 150–160, characterized by an RMSF value of ~ 1 to 3 Å. In this region, residues 150–156 contribute to the formation of a β-sheet, while residues 157–160 form a loop connecting two β-sheets. Interestingly, all mutant structures displayed lower RMSF values (indicating less flexibility and more rigidity) in the 250–270 region, which includes two separate loops and β-sheets. Distinct fluctuations in other regions were also observed. Based on the average RMSF values (Fig. 5B), the mutants can be arranged in descending order: W245G > G270D > L248P > G181S > N196K > F165L > F193V > R247W > D242N > G270C > G270S. Additionally, to assess the spatial distribution and compactness of the folded structure, Rg analysis revealed notably high values for mutants F165L, F193V, G181S, and N196K, suggesting a more extended and less compact structure (Fig. 4C and Fig. S5). The average Rg values for the other four mutants (W245G, G270D, L248P, and R247W) were also significantly higher compared to the wild-type C1q domain (Fig. 5C). These mutants can be arranged in descending order as follows: F165L > F193V > G181S > N196K > W245G > G270D > L248P > R247W > G270S > D242N > G270C.

To further explain the observed instability in specific mutants compared to the native C1q domain, we examined the SASA values (Fig. 4D and Fig. S6). SASA analysis quantifies the exposed surface area of a molecule, with higher values indicating a larger exposed area. Mutants F193V, G270D, F165L, L248P, N196K, and W245G exhibited higher average SASA values compared to the wild-type structure, indicating a greater degree of instability due to increased solvent exposure (Fig. 5D). Conversely, G270C, R247W, and G270S demonstrated lower SASA values. Additionally, an analysis of hydrogen bonds revealed a decrease in the number of hydrogen bonds across all mutated C1q domains (Fig. 4E, F). This decrease likely contributes to their reduced stability and may be associated with increased structural flexibility. Notably, the highest and lowest average scores for H-bond formation were observed in N196K and G270D, respectively (Fig. 5E). Principal Component Analysis (PCA) indicated dominant and positive conformational changes in all five MD parameters exhibited for component 1. However, only three parameters, excluding gyration and hydrogen bonding, displayed positive loadings on component 2 (Fig. 5F). Overall, our findings suggest that mutations in the CTRP6 C1q domain destabilize the protein structure, leading to reduced compactness, increased flexibility due to fewer hydrogen bonds, and greater solvent exposure compared to the native C1q domain.

### Additional analysis

Additional structural changes in the mutant models were elucidated through secondary structure analysis. Our analysis predicted the formation of a new short α-helix in the F193V and G270C variants, as well as the introduction of a β-strand in the D242N and R247W variants at the N-terminal of the CTRP6 C1q domain. Notably, other variants exhibited disruptions in β-sheet length or the elimination of specific short-length sheets within the C1q domain (Fig. S7). In most cases, α-helices and β-sheets were either extended or shortened by one or more residues.

**Fig. 6:**
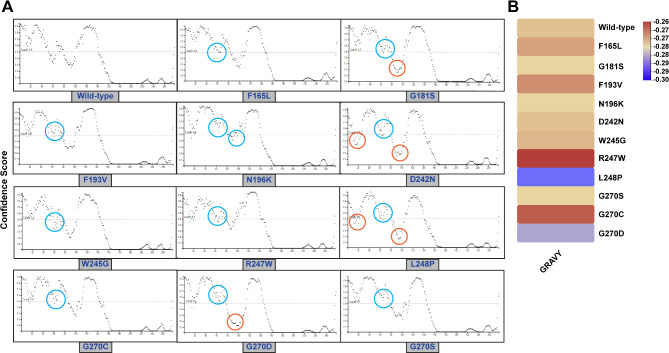
Comparison of structurally disordered regions between wild-type and mutant models, along with an assessment of hydropathicity alteration. **(A)** Blue circles highlight disordered regions, while orange circles represent regions that acquired an ordered structure. **(B)** Increased hydrophilicity (blue) or increased hydrophobicity (red) was predicted in the mutant models compared with wild-type model using GRAVY values.

**Fig. 7:**
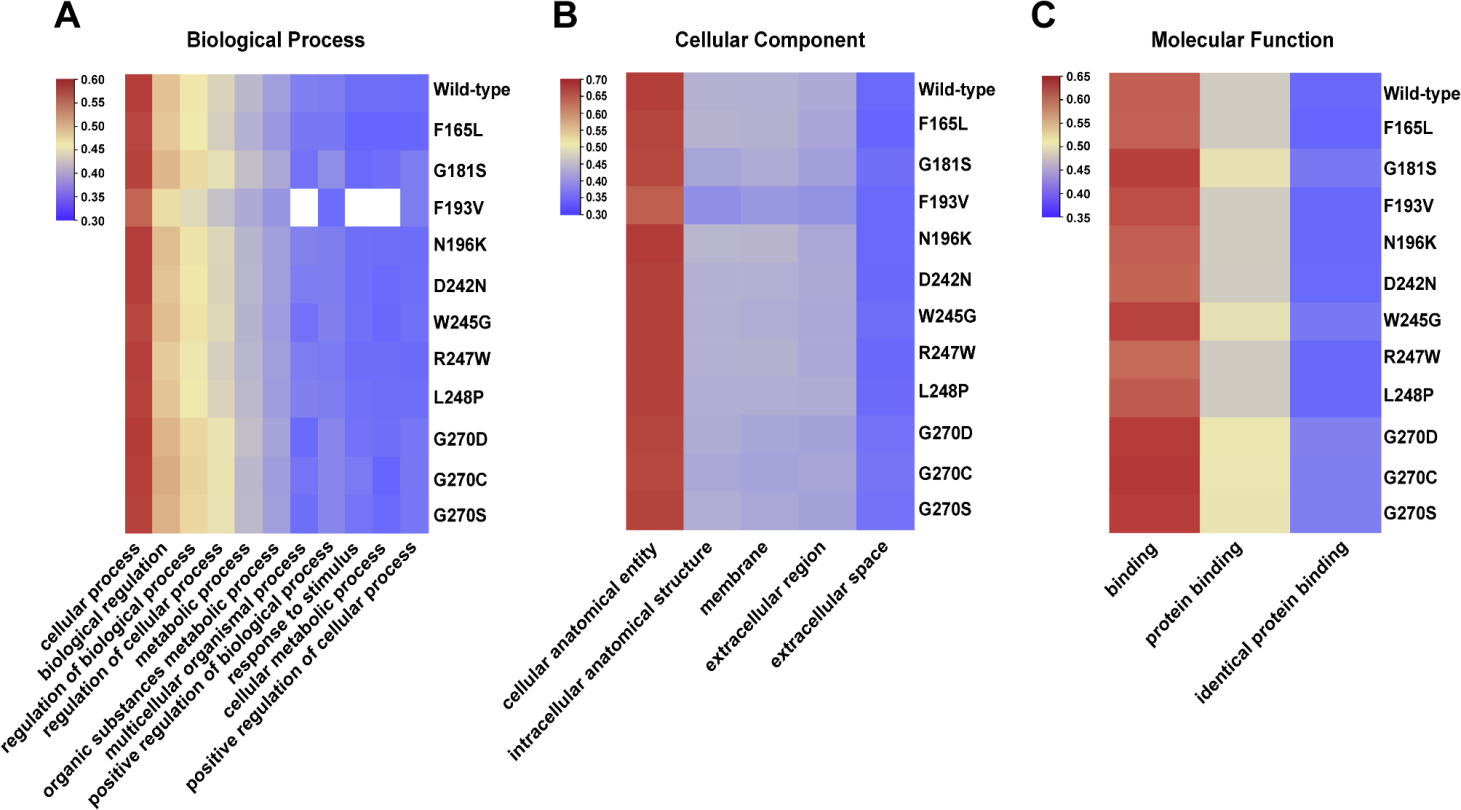
Gene Ontology (GO) terms analysis of biological process (**A**), cellular component (**B**), and molecular function (**C**) for all the deleterious variants.

Mutations can disrupt the structural integrity of proteins, leading to the disordering of specific protein regions. A comparative analysis between damaging variants and the wild-type C1q model revealed significant differences in the disordered regions, with confidence scores exceeding the 0.5 cutoff. While amino acid positions 50–70 in the native C1q domain maintained an ordered structure, this segment became markedly disordered in all mutants, as indicated by the blue circle in Fig. 6A. Some variants (G181S, D242N, L248P, G270D) lowered the confidence scores in other segments, inducing a transition towards a more ordered structure, as highlighted by the orange circle. Moreover, the mutants exhibited a negative GRAVY score, indicative of increased hydrophilicity (Fig. 6B). Notably, the increased hydrophilicity observed in the L248P and G270D mutants might impair the protein’s ability to fold into its native conformation and interact with partner proteins by facilitating interactions with surrounding water molecules, potentially reducing protein stability. Our functional prediction analysis also anticipates potential impacts of these variants on GO terms, including biological processes, molecular functions, and cellular components (Fig. 7**)**.

### Association of missense SNPs with certain types of cancer

**Fig. 8:**
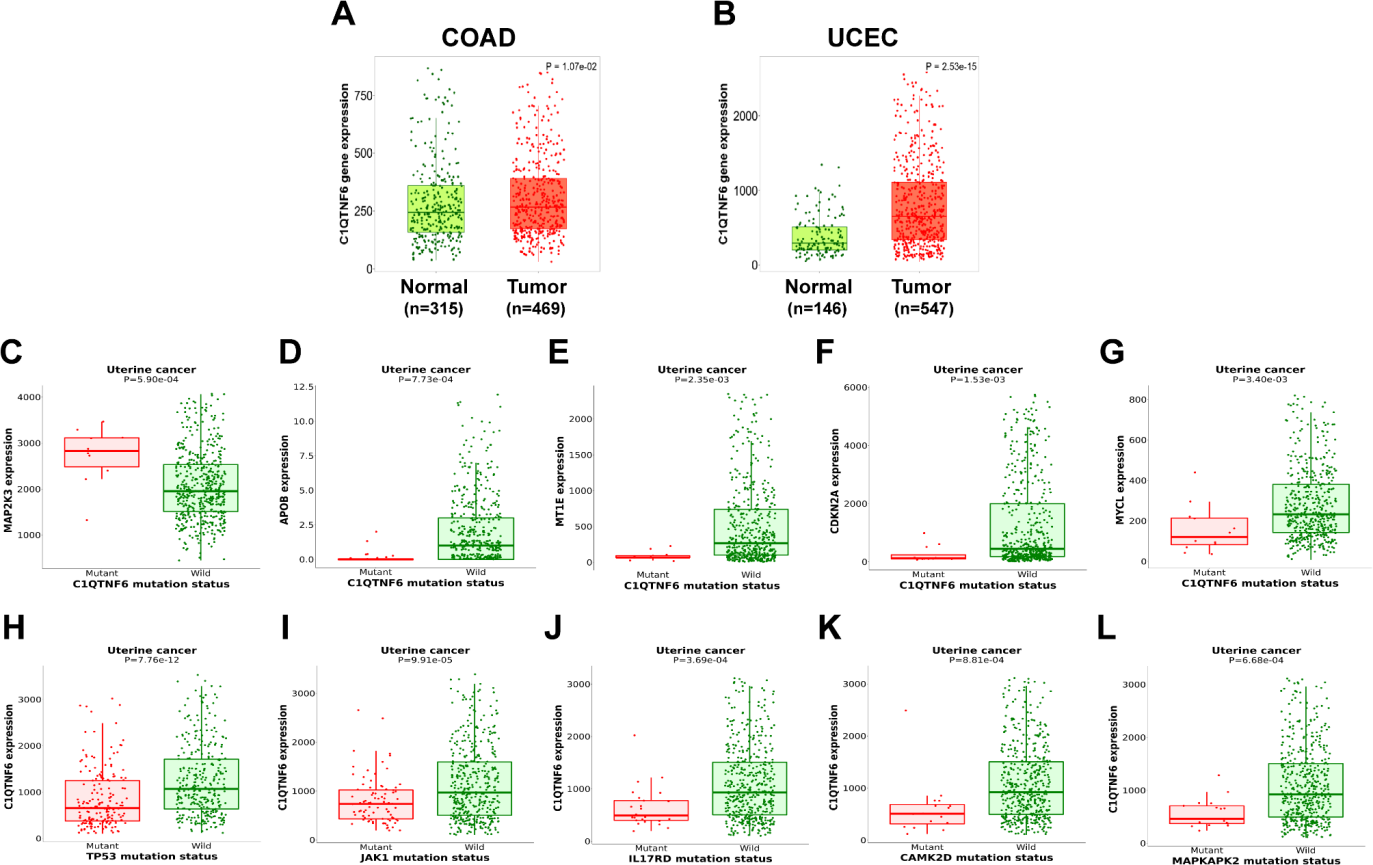
Expression analysis of CTRP6 and its relation to other cancer-related genes in COAD and UCEC. **A**, **B)** Remarkable upregulation of CTRP6 gene expression in both COAD and UCEC patient samples (TNMplot). **C-G)** Upregulated and downregulated top cancer-related genes in genotype analysis (muTarget) using a mutant CTRP6 query. **H-L)** Alterations in CTRP6 expression resulting from several cancer-related genes in mutation status in target analysis (muTarget) using CTRP6 as the target gene.

Based on the mutation profiles from the cBioPortal and canSAR databases, the G181S and R247W variants of CTRP6 were linked to colon adenocarcinoma (COAD) and uterine corpus endometrial carcinoma (UCEC), respectively. Both variants exhibited moderate carcinoma severity with allelic frequencies of 0.19 (G181S) and 0.13 (R247W). The TNMplot database revealed upregulated CTRP6 gene expression in patients with COAD and UCEC, with mean fold changes of 1.16 and 1.91, respectively (Fig. 8A, B). Previous experimental studies have consistently reported CTRP6 overexpression as a contributing factor in the development of various cancers, including hepatocellular carcinoma, gastric cancer, and lung cancer [15]. Our findings suggest that both CTRP6 overexpression and its missense SNPs are involved in the development of COAD and UCEC.

To determine how missense SNPs in the C1q domain of CTRP6 influence the expression of other important cancer-related genes in uterine cancer, we used the CTRP6 gene as a mutant query in the muTarget program (Fig. 8C-G). The ‘genotype’ prediction results revealed a significant upregulation of mitogen-activated protein kinase kinase 3 (MAP2K3) and downregulation of apolipoprotein B (APOB), metallothionein 1E (MT1E), cyclin-dependent kinase inhibitor 2 A (CDKN2A; p16), and MYCL proto-oncogene (MYCL). Additionally, ‘target’ analysis predicted that the mutation status of tumor protein 53 (TP53), Janus kinase 1 (JAK1), interleukin-17 receptor D (IL-17RD), calcium/calmodulin-dependent protein kinase II delta (CAMK2D), and mitogen-activated protein kinase-activated protein kinase 2 (MAPKAPK2) significantly reduced the expression of CTRP6 in uterine cancer (Fig. 8H-L). These genes play direct roles in cellular mechanisms associated with cancer and can contribute to its progression. These comparative results suggest that CTRP6 mutations may modulate the expression of key genes, potentially influencing cancer development.

## Discussion

In this study, we utilized various pathogenicity prediction algorithms to discern 11 deleterious mutations within the highly conserved C1q domain of human CTRP6. We systematically characterized the effects of these mutations on the stability, flexibility, structural conformation, compactness, stiffness, and overall functionality of the protein. Notably, our findings revealed that two mutations (G181S and R247W) are specifically associated with COAD and UCEC, respectively. This study not only enhances our understanding of the functional consequences of CTRP6 mutations but also offers valuable insights into the mutations most likely involved in cancer initiation and development, potentially streamlining future research efforts.

GWAS have successfully identified variants associated with various diseases by primarily focusing on common variants that are prevalent in a significant portion of the population. While these common variants provide valuable insights into the genetic basis of various traits, population genetics, and disease susceptibility, the study of rare variants—those present in a smaller percentage of the population—is crucial for understanding complex human diseases. Rare variants, particularly those that are deleterious, can have a more significant impact on gene function and expression [80, 81]. Certainly, not all rare variants are harmful, as some may have no impact on protein function. However, assessing the risk associated with rare variants is challenging and often requiring individual sequencing data analysis—unlike common variants, which are easier to identify. In contrast to GWAS, our in-silico approach specifically targets rare missense SNPs. We identified 11 deleterious mutations within the highly conserved C1q domain of human CTRP6 using pathogenicity prediction algorithms. This study contributes to streamlining the need for extensive mutant studies of CTRP6 in cancer by pinpointing the most deleterious mutations likely associated with different cancer types.

In our study, we characterized the structural and functional impact of 11 missense SNPs (F165L, G181S, F193V, N196K, D242N, W245G, R247W, L248P, G270S, G270C, G270D) within the CTRP6 C1q domain. Given the evolutionary conservation observed across diverse species, substitutions at these positions—especially those involving changes between neutral and charged residues, or the replacement of a positively charged residue with a negatively charged one—are likely to have a significant impact on protein structure and function. Notably, non-polar residues at positions F165, F193, W245, and L248 are substituted with other non-polar residues, while the native non-polar residues at G181 and G270 (G270S/C, not G270D) are replaced by polar residues. Other specific substitutions include N196K (polar to positively charged), D242N (negatively charged to polar), R247W (positively charged to non-polar), and G270D (non-polar to negatively charged). Residues F165, F193, W245, L248, R247, and G270 form several hydrogen, hydrophobic, and electrostatic bonds with neighboring residues in the core region of the C1q structure (Table S3). Substitutions at these positions result in reduced stability of the hydrophobic core of the CTRP6 C1q domain. This is due to decreased compactness and increased solvent accessibility, along with the disordering of other structural components, as shown by our RMSF, Rg, and SASA results from MD simulations. Notably, G181, N196, and D242 are located on the surface of the C1q structure, and their substitutions do not significantly alter the solvent accessibility of the C1q domain, particularly in the cases of G181S and D242N, as indicated by average SASA values. However, the substitution of G181 is particularly critical due to its small side chain and its role in regions such as β-turns or loop areas, where flexibility is crucial for preserving the native protein folding.

**Fig. 9:**
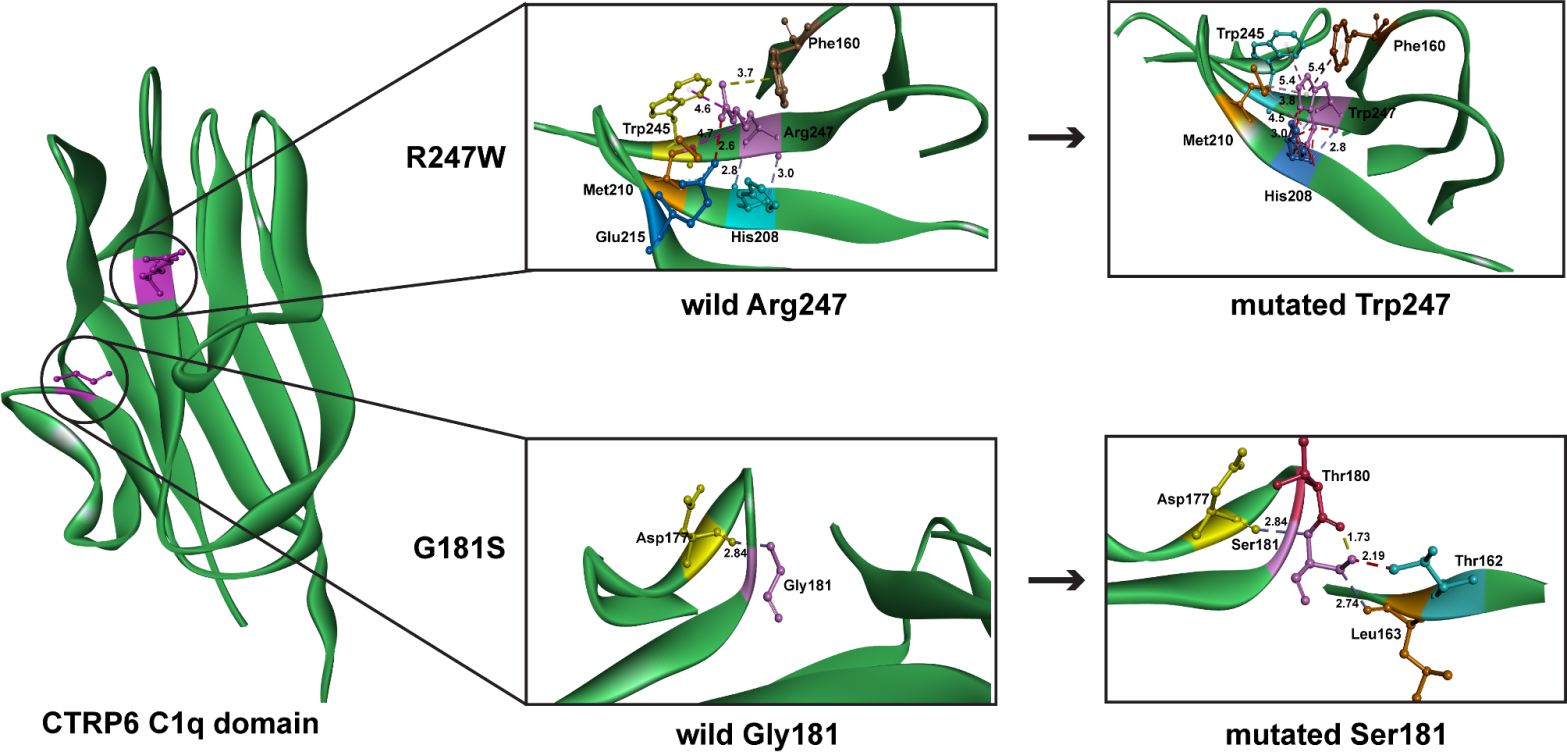
Structural analysis of G181S and R247W mutations. Illustration of broken and newly formed bonds after substitution, with the distance of each bond represented.

Notably, the mutations G181S, R253C, and R247W are specifically associated with colon adenocarcinoma, prostate adenocarcinoma, and uterine corpus endometrial carcinoma, respectively. These three mutations are the only ones identified to have a clear association with cancer in both the cBioPortal and canSAR databases. However, R253 was excluded from the evolutionary conservation analysis due to its conservation score of 7, indicating a lower degree of evolutionary conservation compared to other residues. G181 is situated within a small loop, accompanied by Ala179 and Thr170, all of which possess small side chains, making them well-suited for loops that contribute to the structural flexibility of the protein. Additionally, G181 forms a critical hydrogen bond with Asp177, which plays a stabilizing role in the loop without imparting excessive rigidity or flexibility. Substituting G181 with the polar and hydrophilic serine residue introduces an additional hydrogen bond with Leu163, potentially leading to an undesirable increase in loop rigidity (Fig. 9). These observations are consistent with our flexibility and RMSF analysis results, which show that the G181S substitution induces increased rigidity in various regions of the C1q domain, particularly within the 150–265 amino acid segment.

The substitution of the conserved arginine (R247) with tryptophan in the CTRP6 C1q domain is implicated in causing moderate UCEC. Arginine, a positively charged residue, is often found in active sites and plays a crucial role in protein-protein interactions and the formation of salt bridges. In the CTRP6 C1q domain, R247 is located within a β-sheet and engages in several interactions with neighboring residues. These interactions include two conventional hydrogen bonds with His208, a salt bridge hydrogen bond with Glu215, two hydrophobic interactions with Met210 and Trp245, and electrostatic bonding with Phe160 (Fig. 9). In the R247W substitution, the introduction of tryptophan (Trp247) disrupts the critical salt bridge hydrogen bond with Glu215. Salt bridges are essential for maintaining the overall stability of protein structures and substrate binding. The mutation from arginine to tryptophan not only disrupts this salt bridge but also results in the loss of positively charged residues, replacing them with a bulky aromatic side chain. This alteration can significantly impact the stability of the β-sheet, potentially leading to structural changes that balance increased rigidity and flexibility in other segments of the protein, as indicated by our flexibility analysis.

CTRP6 is most highly expressed in the endometrium, as shown by data from the Human Protein Atlas. Mutations in CTRP6 have been predicted to cause significant changes in the expression of several cancer-related genes in uterine cancer. One notable example is the upregulation of MAP2K3, a kinase involved in the MAPK14/p38-MAPK signaling pathway. This pathway is critical in cellular responses to stress and inflammation, and its dysregulation can contribute to cancer progression. The upregulation of MAP2K3 due to CTRP6 mutations may promote oncogenic transformation in primary cells, as MAP2K3 is known to support tumor cell survival and proliferation [82]. This suggests that CTRP6 mutations could play a crucial role in driving cancer development in the endometrium through the modulation of key signaling pathways like MAPK. Another critical gene impacted by CTRP6 mutations is CDKN2A (p16), which functions as a tumor suppressor by regulating cyclin-dependent kinases to prevent uncontrolled cell division and inhibit cell proliferation [83]. The significant downregulation of CDKN2A in uterine cancer due to CTRP6 mutations may contribute to uncontrolled cell division, promoting tumor growth. In addition, Metallothionein-1E (MT1E), a gene responsible for regulating metal levels in the body, is also notably downregulated by CTRP6 mutations. MT1E has been implicated in various cancers, with studies revealing its downregulation and potential role in liver and prostate cancer [84, 85]. The reduction in MT1E expression in uterine cancer may further contribute to the progression and severity of the disease. Apolipoprotein B (APOB), a key structural component of plasma lipoproteins involved in lipid metabolism and transport, has recently been implicated in the onset of various human cancers [86]. While increased expression of APOB has been observed in breast cancer, bladder cancer, and ovarian cancer, it is downregulated in other cancer types, including hepatocellular cancer, colorectal cancer, and gastric cancer. In the context of CTRP6 mutations, the significantly reduced expression of APOB may be linked to the development of uterine cancer. The downregulation of APOB could potentially disrupt lipid metabolism and transport, contributing to the metabolic alterations often observed in cancer cells and promoting tumor progression in uterine cancer.

When CTRP6 was used as a ‘target’ gene in the muTarget analysis, it was revealed that certain mutations in other cancer-related genes, such as TP53, JAK1, IL-17RD, and MAPKAPK2, led to a significant reduction in CTRP6 expression in uterine cancer. This finding suggests that variants in one tumor-related gene can impact the expression of another gene within the same pathway or category, potentially exacerbating the tumor’s condition. TP53 mutations, commonly observed in numerous cancers, play a critical role in tumorigenesis and are detected in approximately 25% of all endometrial cancer patients [87]. The reduction in CTRP6 expression, along with the presence of TP53 mutations, suggests that CTRP6 may play a role in the development of specific subtypes of uterine cancer, especially those that do not have TP53 mutations. Additionally, mutations in JAK1 and MAPKAPK2 have been associated with various types of cancer [88, 89], but their relationship with CTRP6 expression remains to be fully explored. Understanding these gene-gene interactions could help identify potential biomarkers and therapeutic targets for cancer treatment.

CTRP6 has been implicated in multiple cancer types, such as oral squamous cell carcinoma, gastric cancer, lung cancer, bladder cancer, head and neck squamous cell carcinoma, breast cancer, and clear cell renal cell carcinoma [11, 15, 16]. Its upregulation has been linked to several critical cellular processes, including cell viability, proliferation, apoptosis, growth, invasion, migration, and cell cycle regulation. The association between 2 out of the 11 deleterious missense SNPs with COAD and UCEC further validates our in-silico methodology. It is important to note that our findings are based solely on computational predictions, and the lack of experimental validation limits their applicability in clinical contexts. To substantiate these results, future work should focus on experimentally validating the 11 missense variants through in vitro and in vivo studies. Functional assays, including protein structural analysis, interaction studies, and activity assays, are essential to confirm their biological significance. Additionally, exploring the association of these variants with specific diseases in population studies could further illuminate their clinical relevance.

## Conclusions

In conclusion, our comprehensive study elucidates the structural and functional impact of the 11 most deleterious SNPs within the CTRP6 C1q domain using computational tools. These SNPs induce significant fluctuations in the stability, compactness, solvent accessibility, and disorderliness of the C1q domain structure compared to the wild-type protein model. Building on the established correlation between CTRP6 upregulation and various cancer types, we further demonstrate that the G181S and R247W mutations in the C1q domain are associated with COAD and UCEC, respectively. Furthermore, our study reveals insights into additional cancer-related genes, whose expression changes are attributed to mutated CTRP6. The underlying mechanisms governing these alterations will be elucidated in future investigations.

## Electronic supplementary material

Below is the link to the electronic supplementary material.

Supplementary Material 1

Supplementary Material 2

## Data Availability

All data generated or analyzed during this study are included in this published article and its supplementary information files.

## Abbreviations

CTRPs: C1q/TNF-related proteins
SNPs: Single nucleotide polymorphisms
MD: Molecular dynamics
RMSD: Root-mean-square deviation
RMSF: Root-mean-square fluctuation
Rg: Radius of gyration
SASA: Solvent-accessible surface area
GRAVY: Grand average of hydropathy
GO: Gene Ontology
COAD: Colon adenocarcinoma
UCEC: Uterine corpus endometrial carcinoma
