# Supplementary material for: Comprehensive characterization of pathogenic missense CTRP6 variants and their association with cancer
Source: BMC Cancer. 2025 Feb 20;25:304. doi: 10.1186/s12885-025-13685-0 (PMC11840981; doi:10.1186/s12885-025-13685-0)

**Fig. S1** Distribution of non-coding and coding variants of human CTRP6.

**Fig. S2** Multiple sequence alignment of the C1q domain of CTRP6 across diverse vertebrate taxa, including mammals, fishes, and birds, generated using ClustalW. Nine highly-conserved amino acid residues with predicted deleterious substitutions are marked by arrows and rectangular boxes.


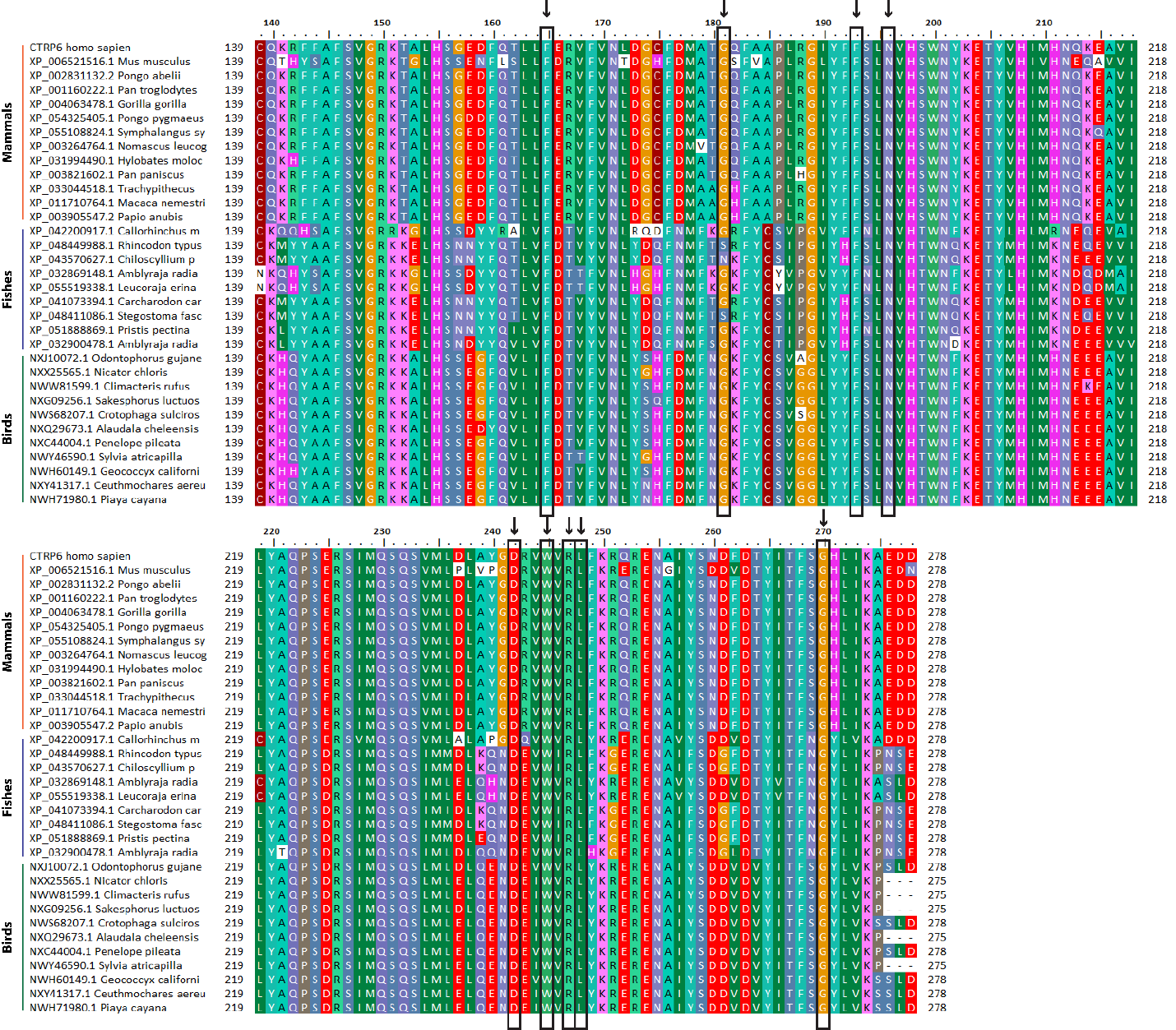


**Fig. S3** Comparison of root mean square deviation (RMSD) between wild-type and mutated structures.

| **RMSD** | | |
| --- | --- | --- |
| 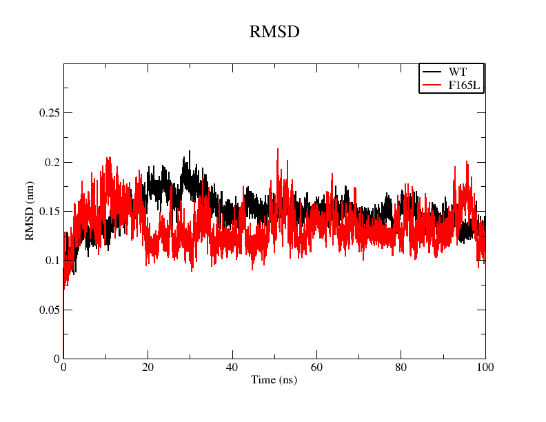 | 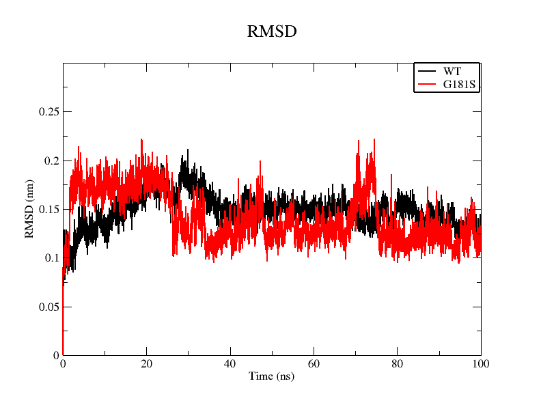 | 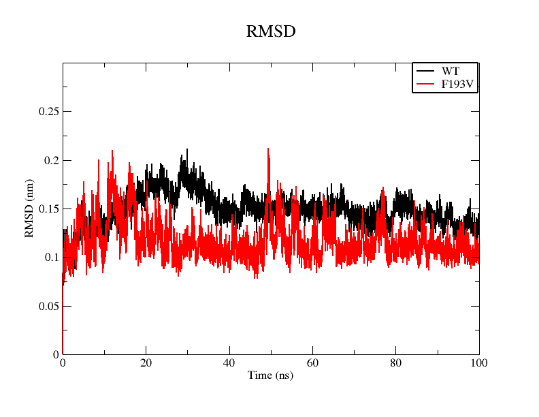 |
| **F165L** | **G181S** | **F193V** |
| 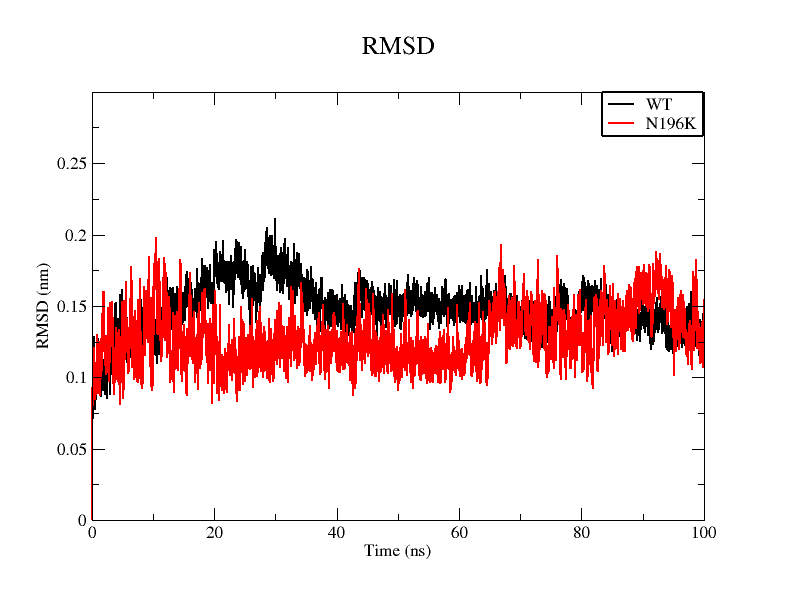 | 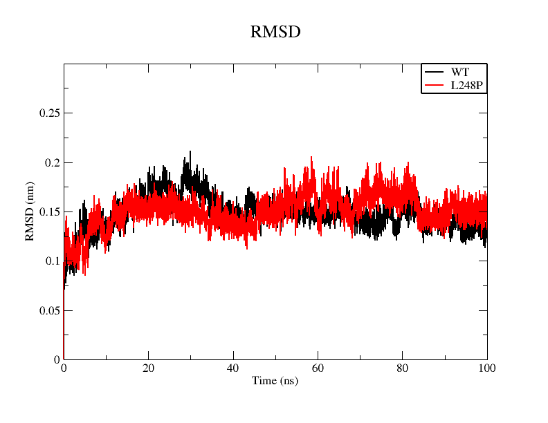 | 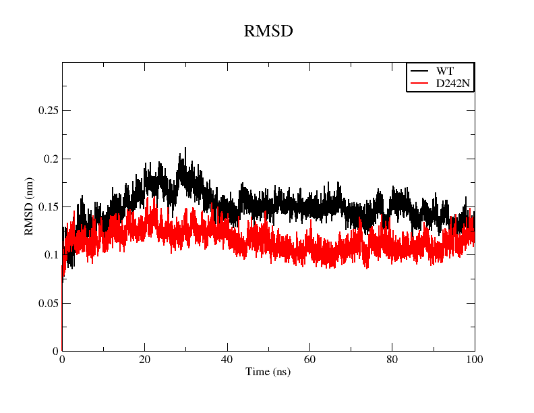 |
| **N196K** | **L248P** | **D242N** |
| 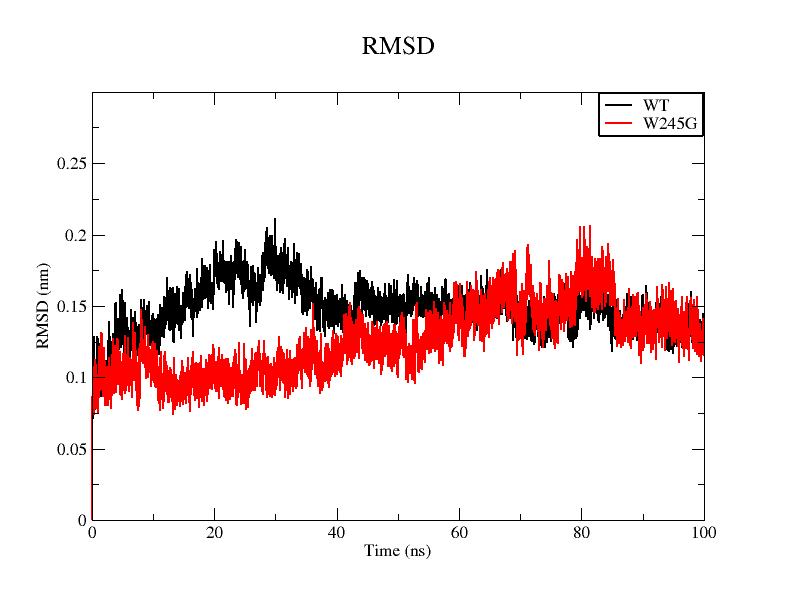 | 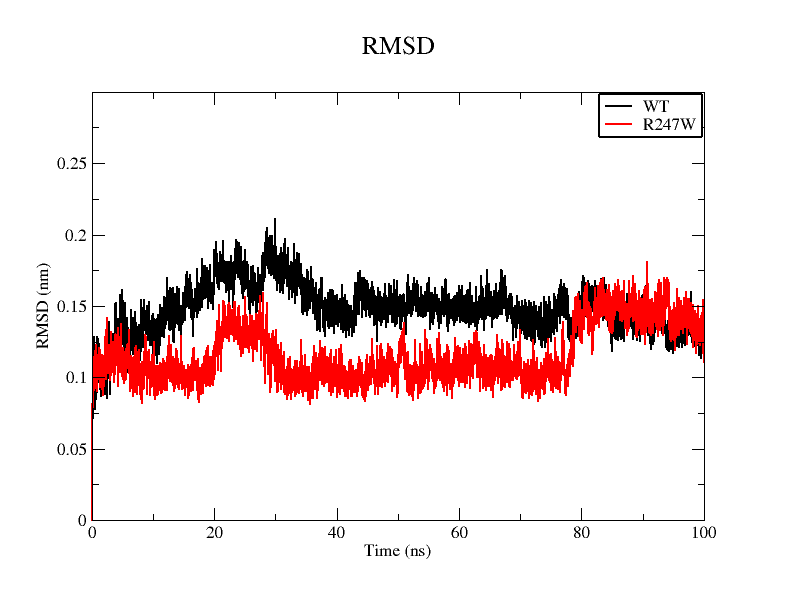 | 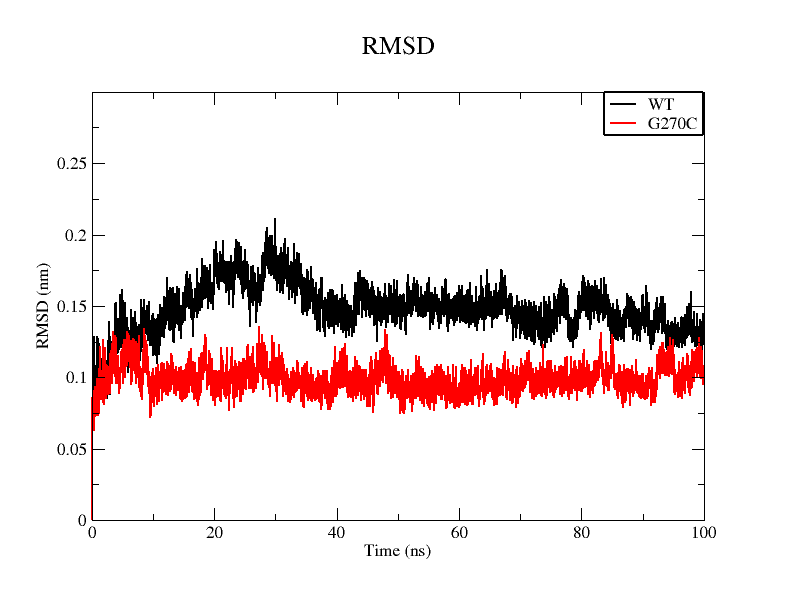 |
| **W245G** | **R247W** | **G270C** |
| 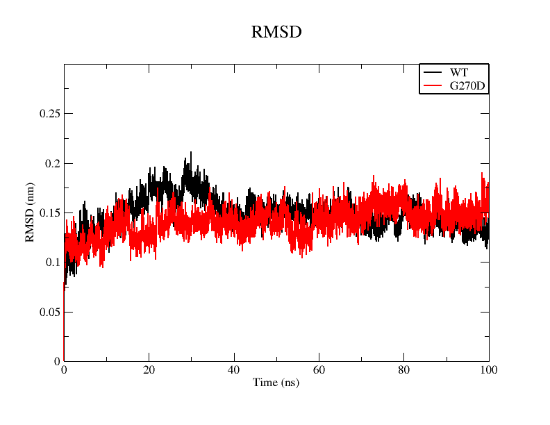 | 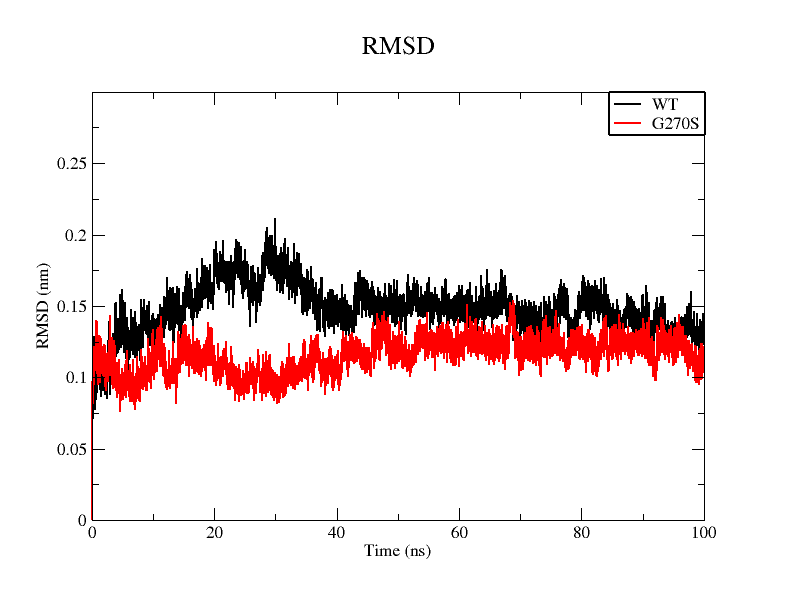 |  |
| **G270D** | **G270S** |  |

**Fig. S4** Comparison of root mean square fluctuation (RMSF) between wild-type and mutated structures.

| **RMSF** | | |
| --- | --- | --- |
| 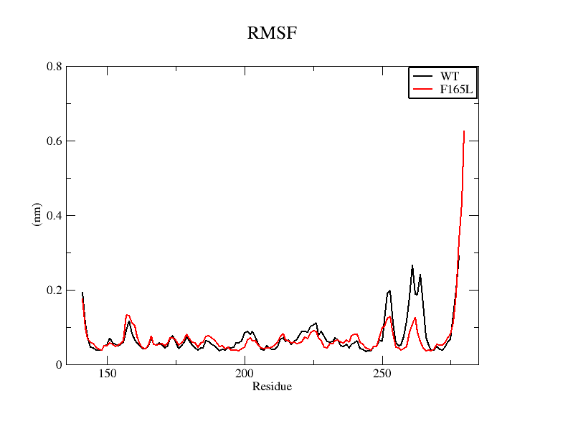 | 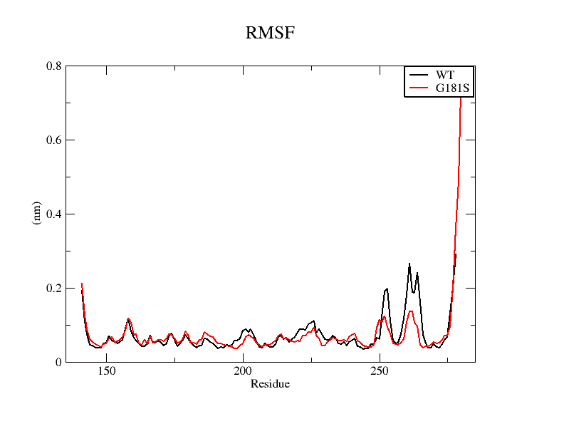 | 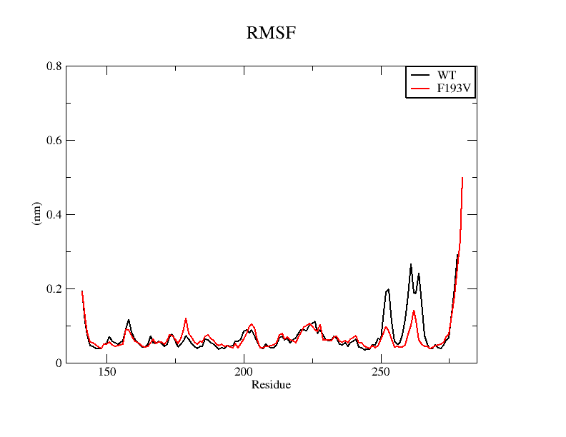 |
| **F165L** | **G181S** | **F193V** |
| 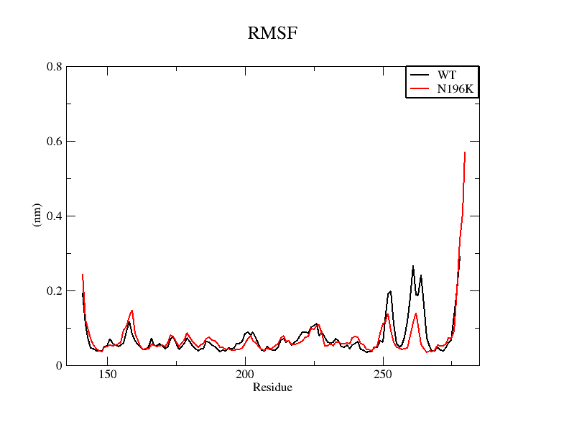 | 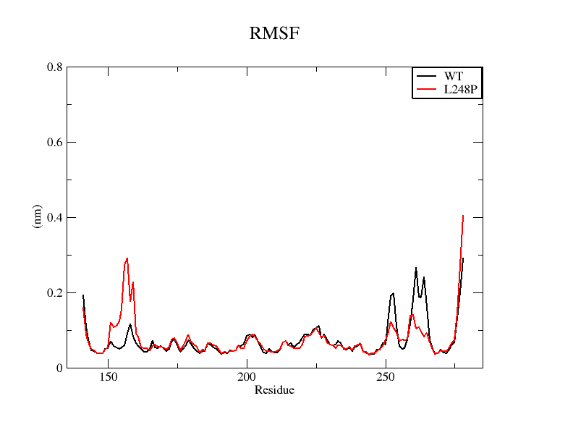 | 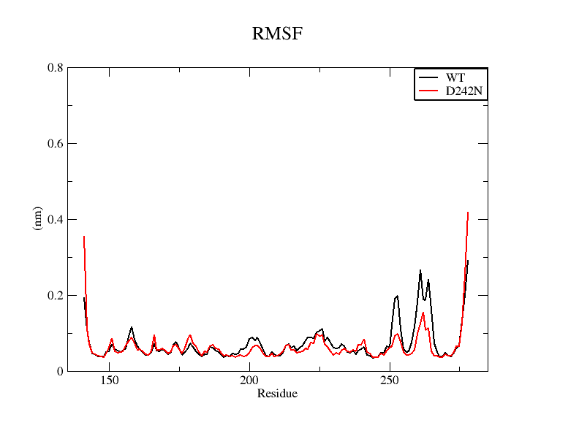 |
| **N196K** | **L248P** | **D242N** |
| 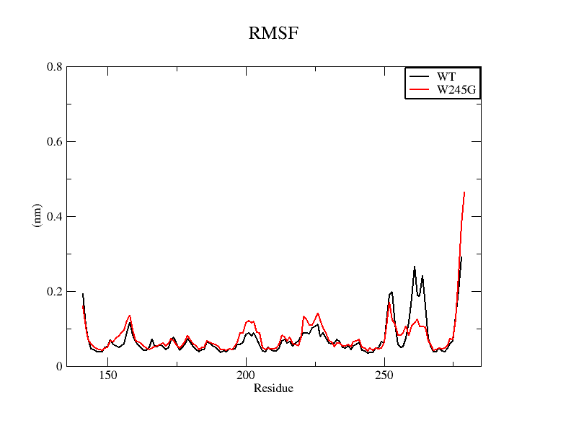 | 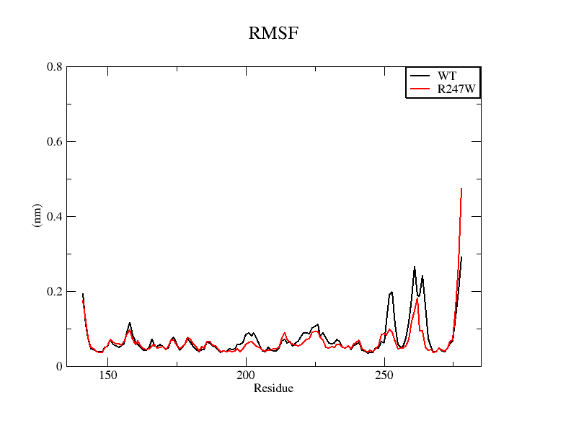 | 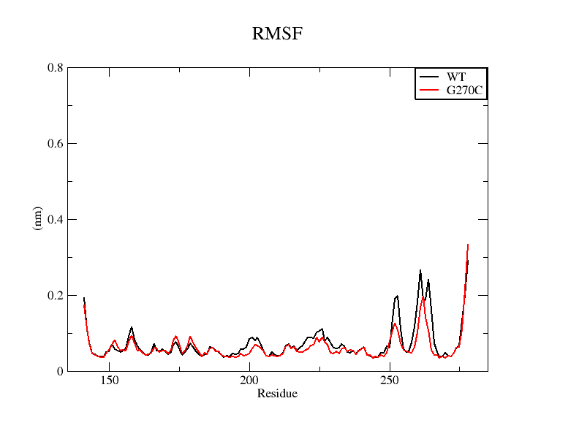 |
| **W245G** | **R247W** | **G270C** |
| 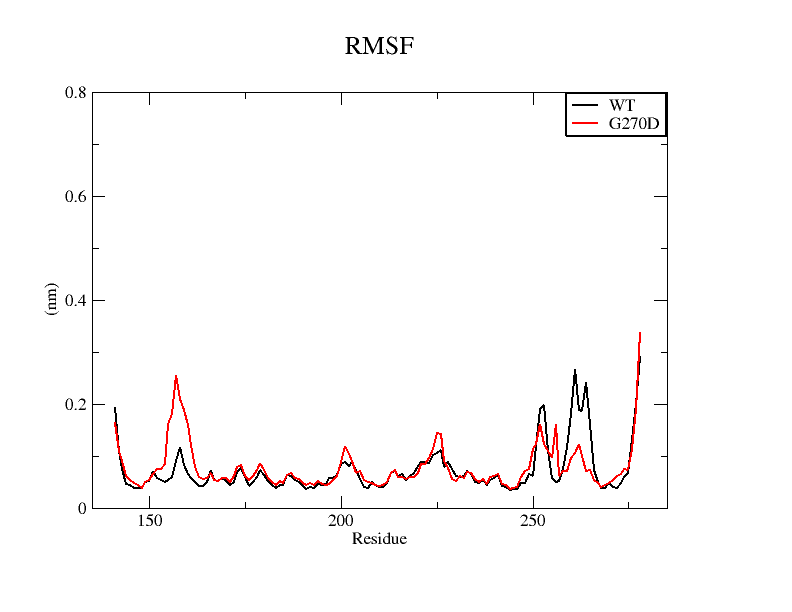 | 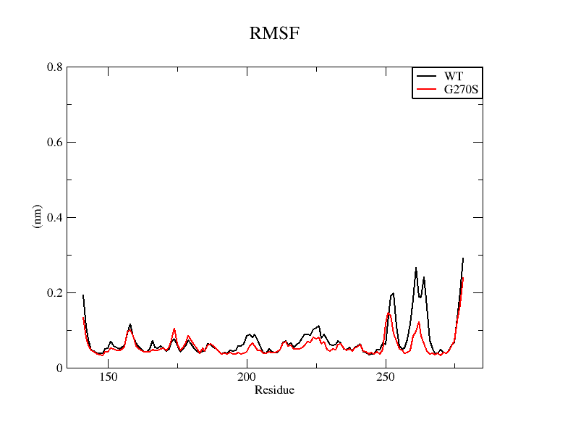 |  |
| **G270D** | **G270S** |  |

**Fig. S5** Comparison of radius of gyration (Rg) between wild-type and mutated structures.

| **Radius of gyration** | | |
| --- | --- | --- |
| 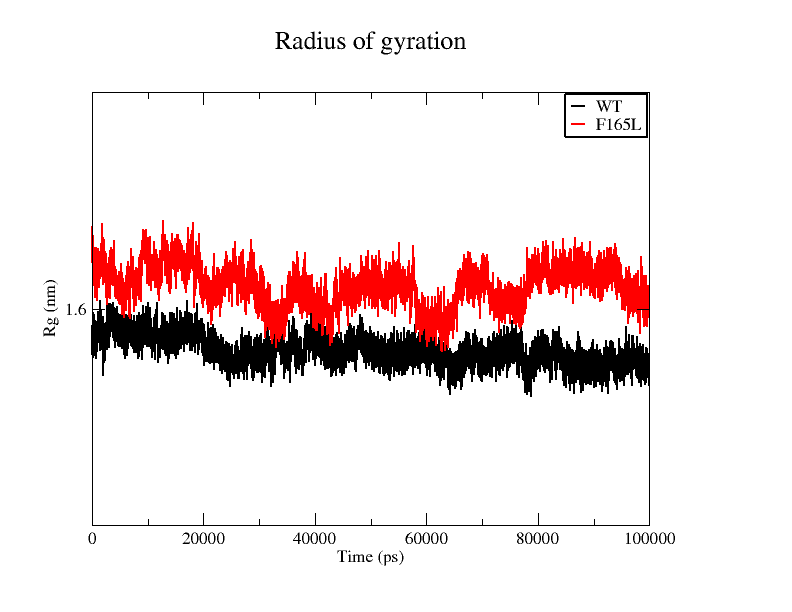 | 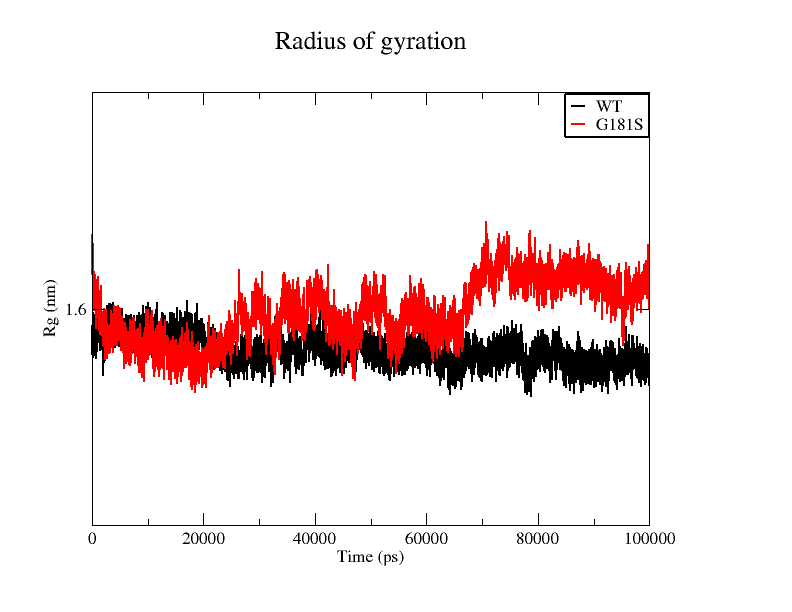 | 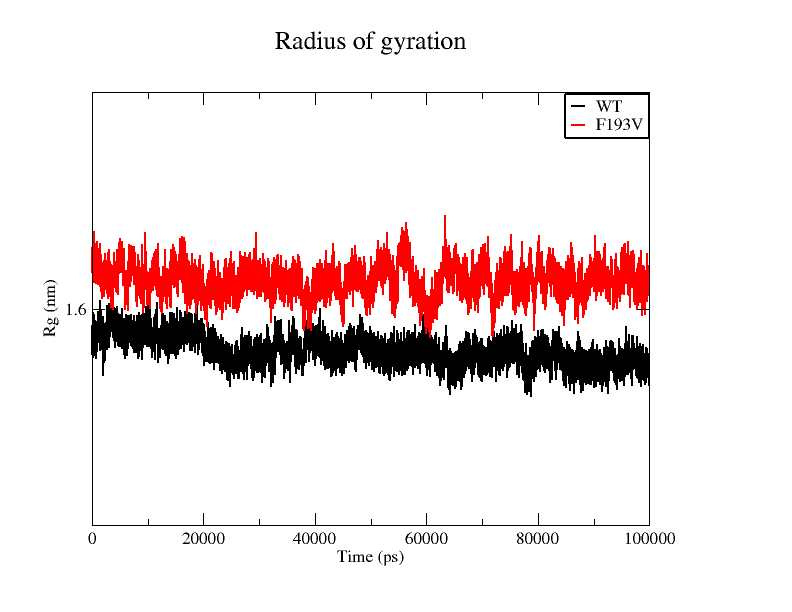 |
| **F165L** | **G181S** | **F193V** |
| 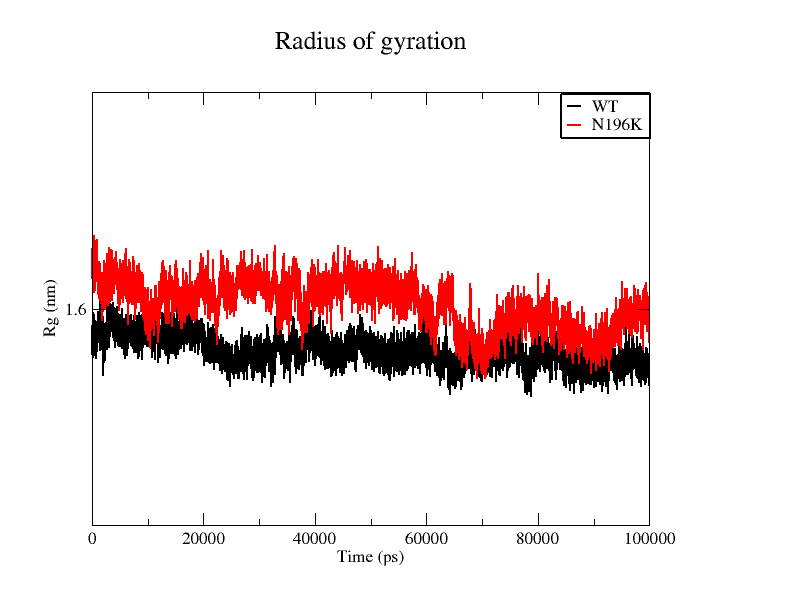 | 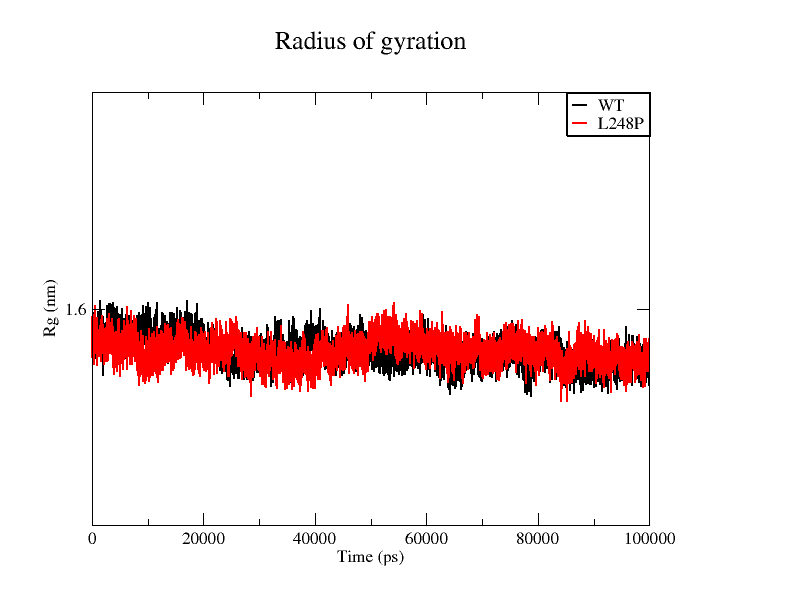 | 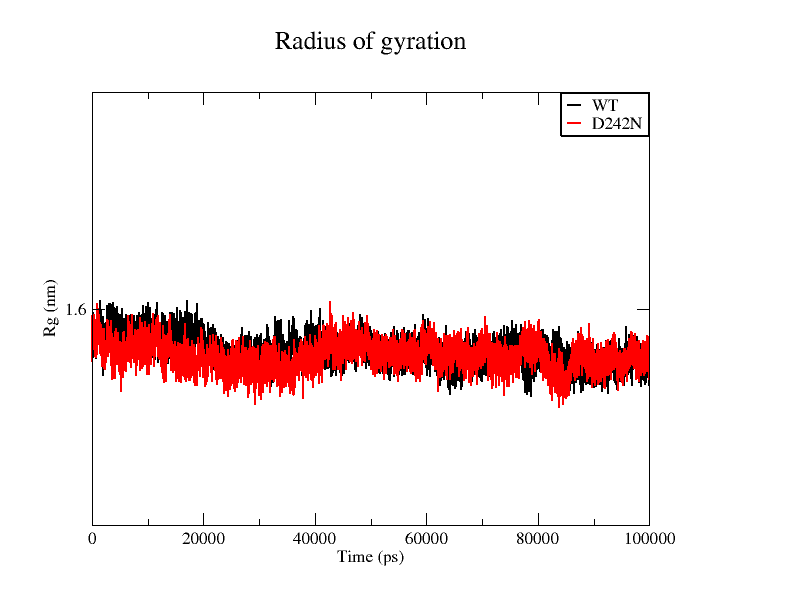 |
| **N196K** | **L248P** | **D242N** |
| 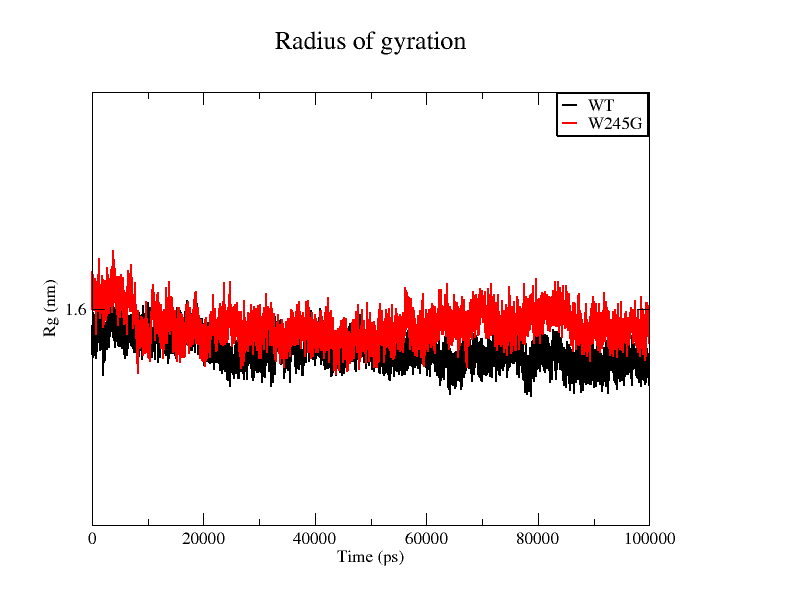 | 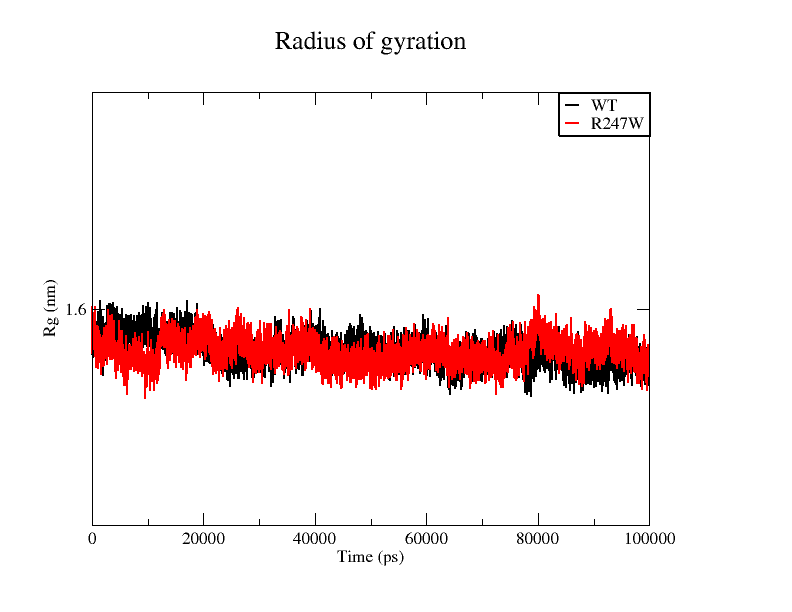 | 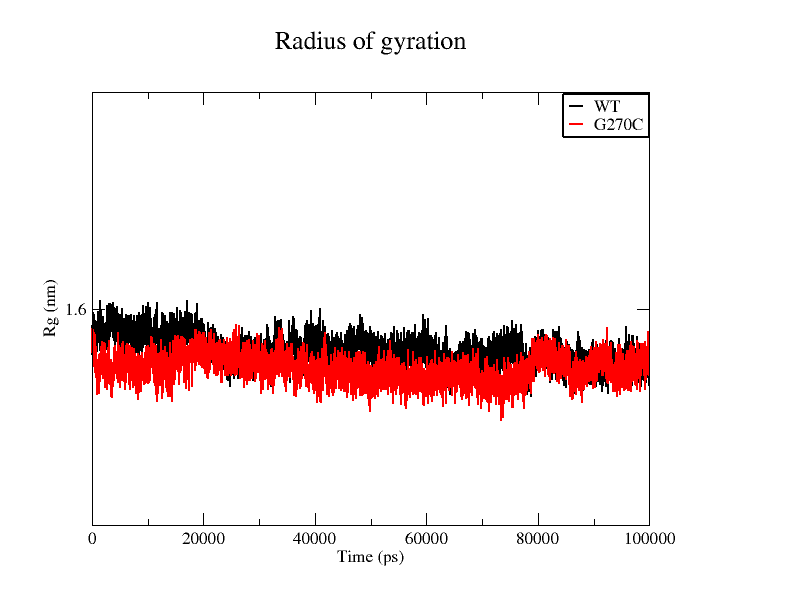 |
| **W245G** | **R247W** | **G270C** |
| 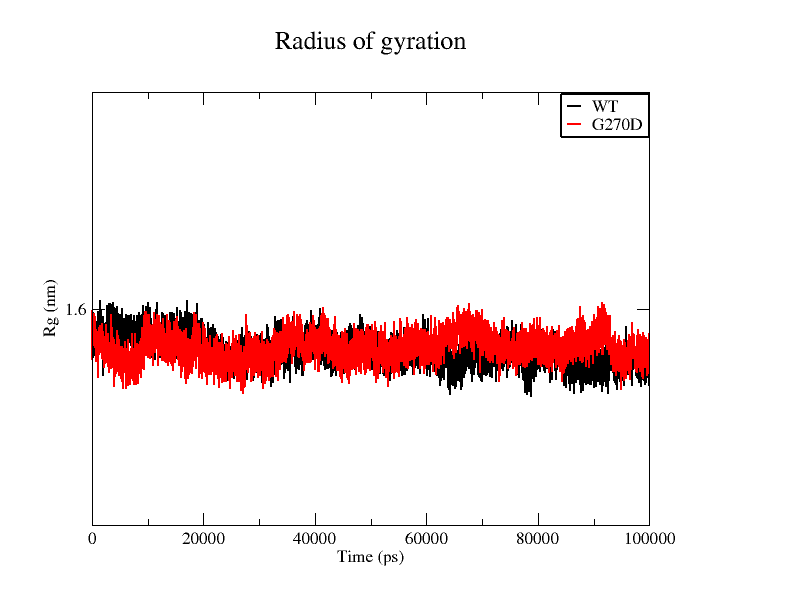 | 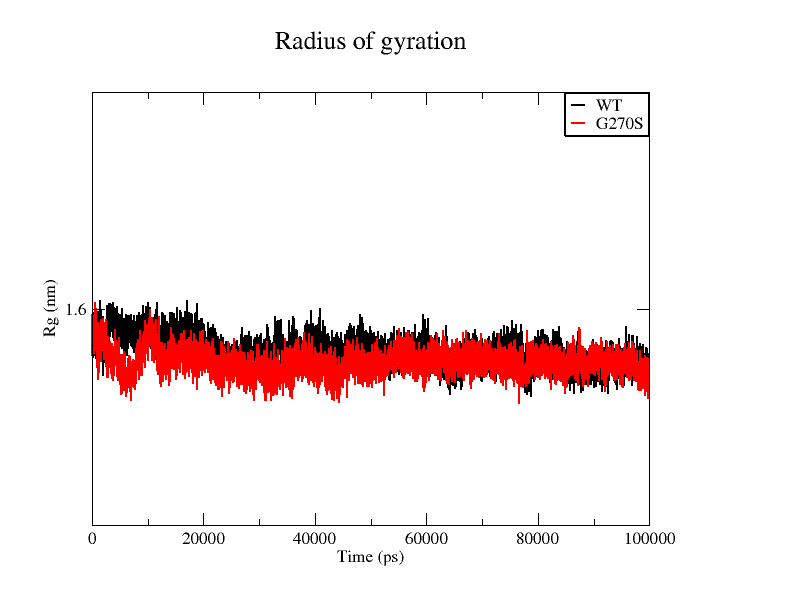 |  |
| **G270D** | **G270S** |  |

**Fig. S6** Comparison of solvent accessible surface area (SASA) between wild-type and mutated structures.

| **SASA** | | |
| --- | --- | --- |
| 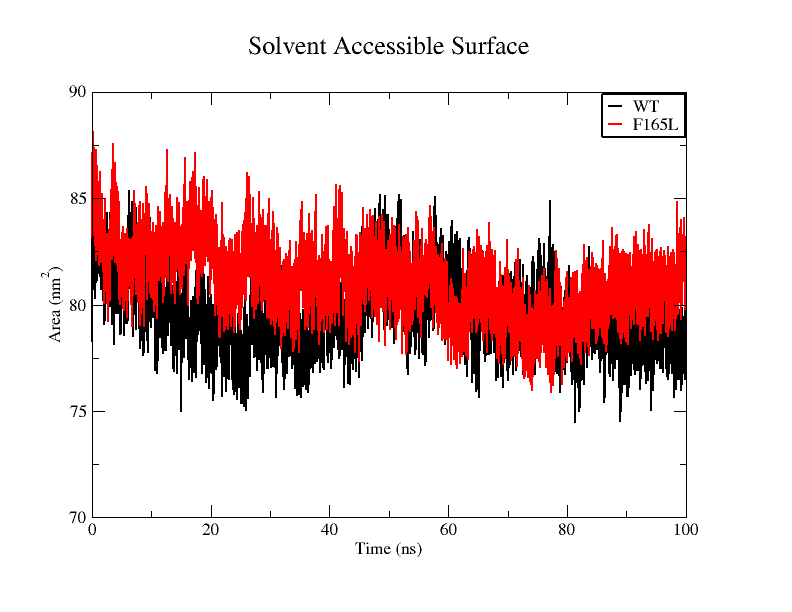 | 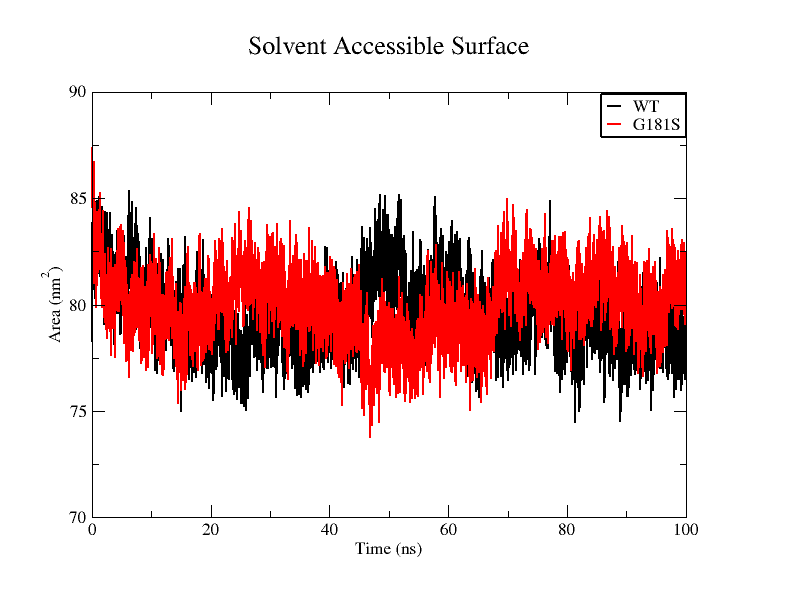 | 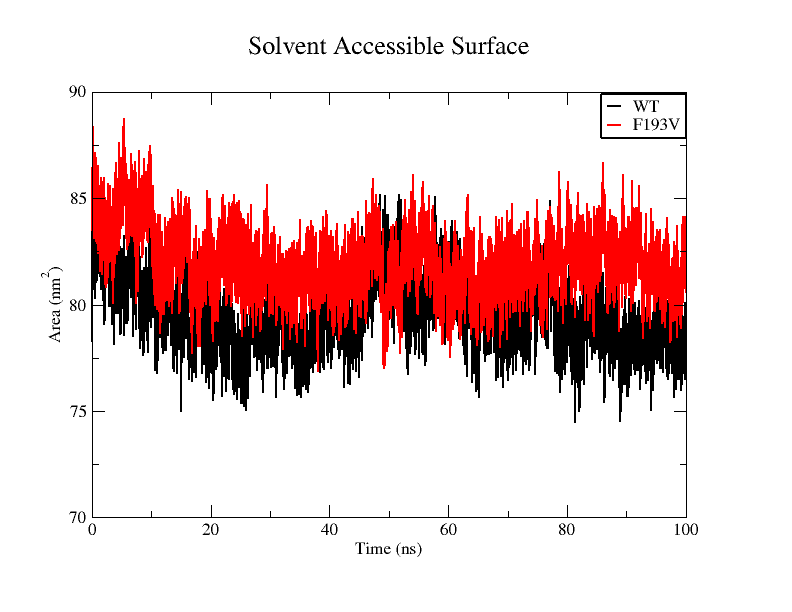 |
| **F165L** | **G181S** | **F193V** |
| 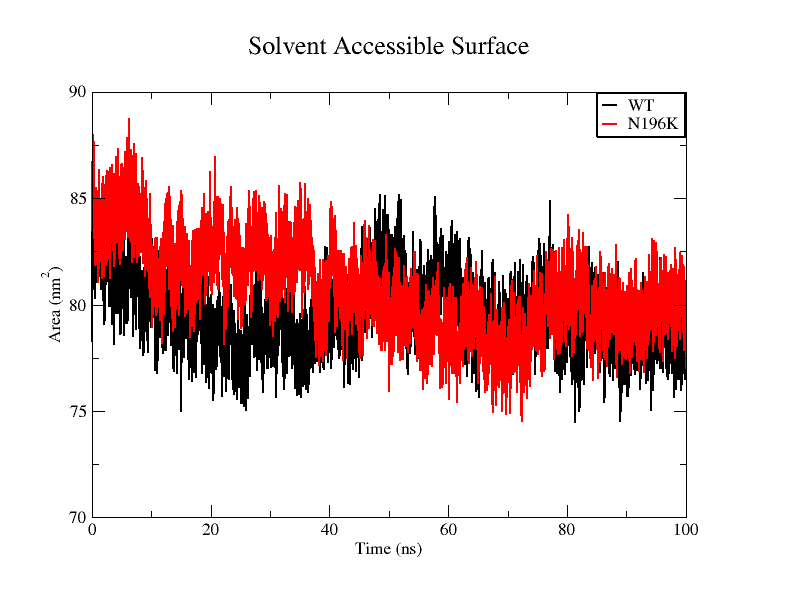 | 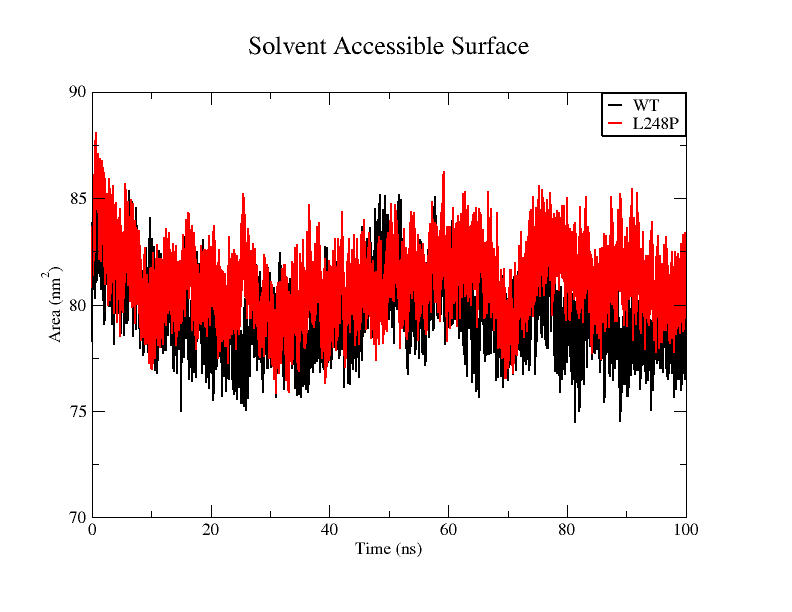 | 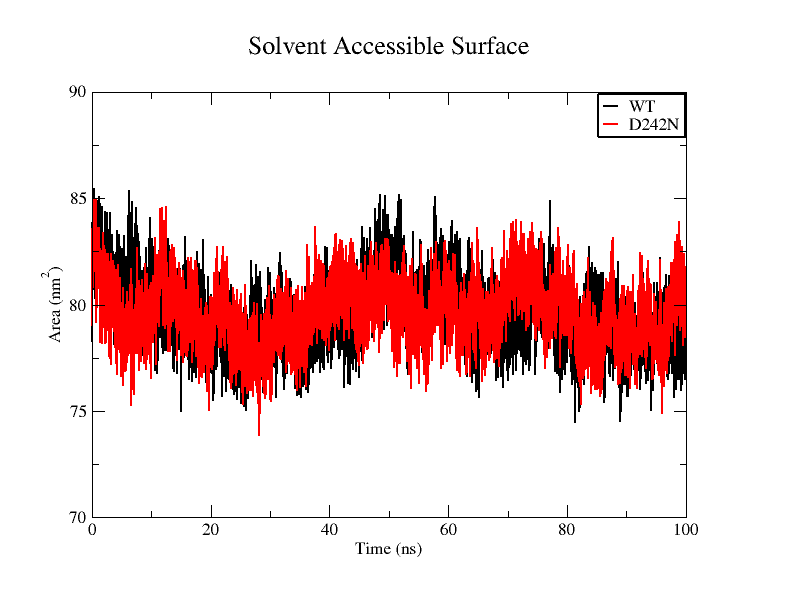 |
| **N196K** | **L248P** | **D242N** |
| 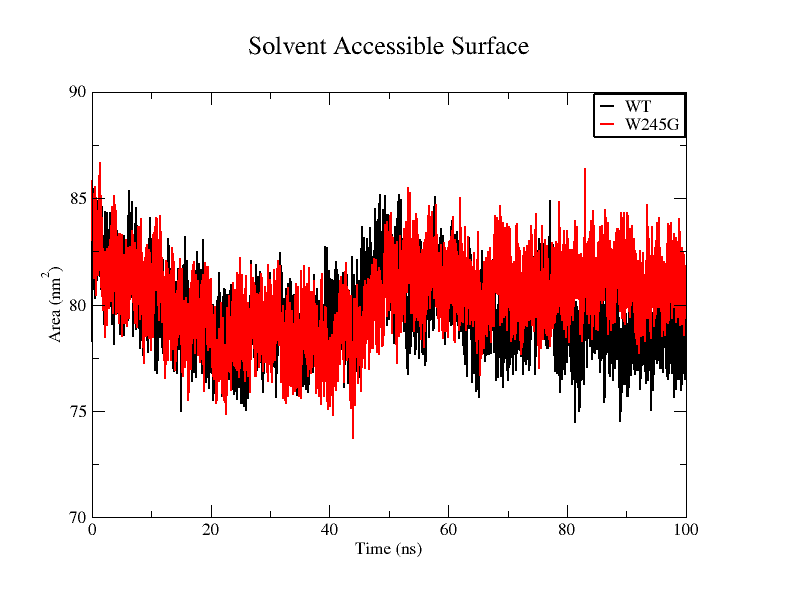 | 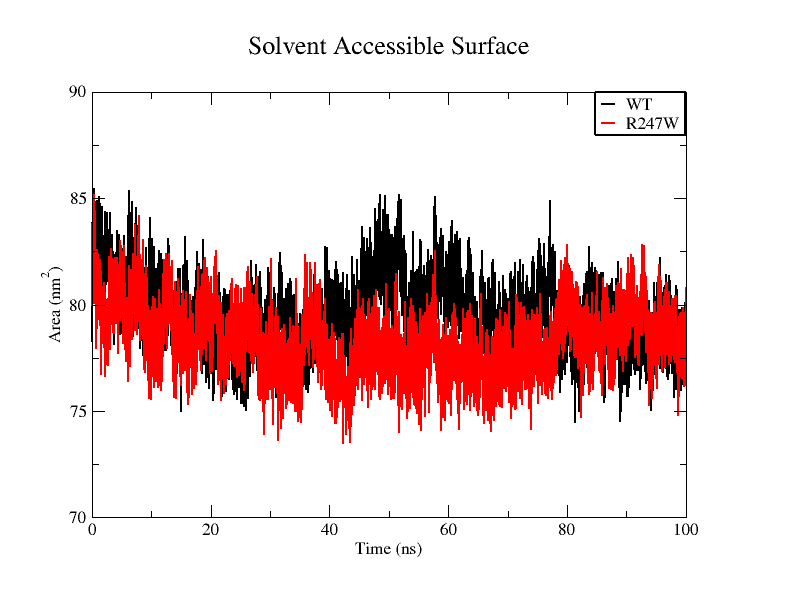 | 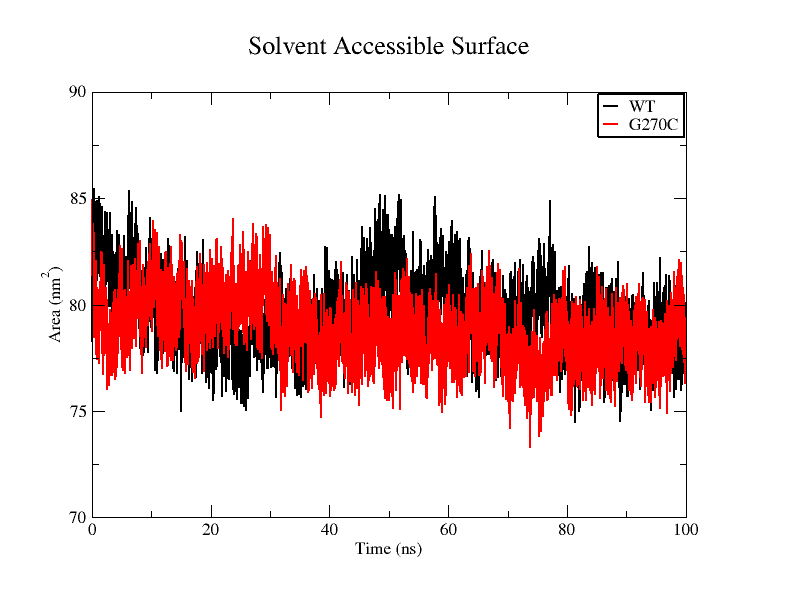 |
| **W245G** | **R247W** | **G270C** |
| 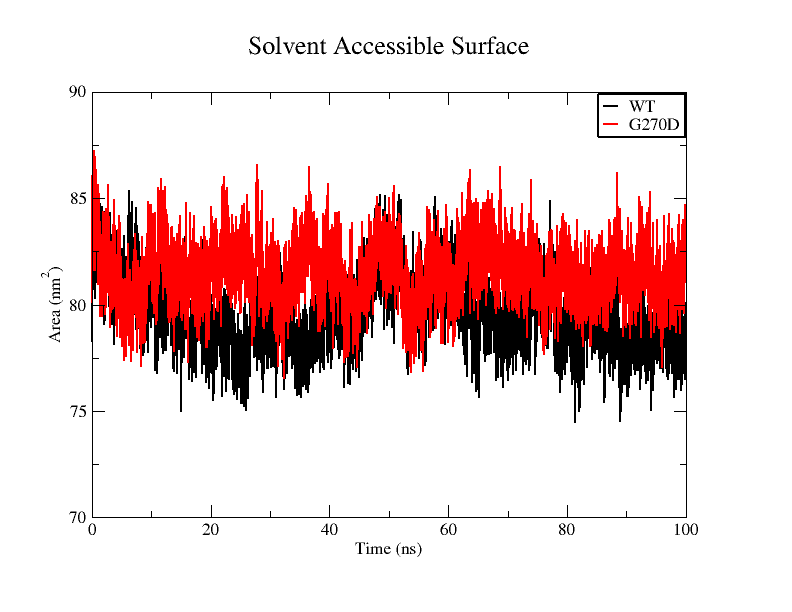 | 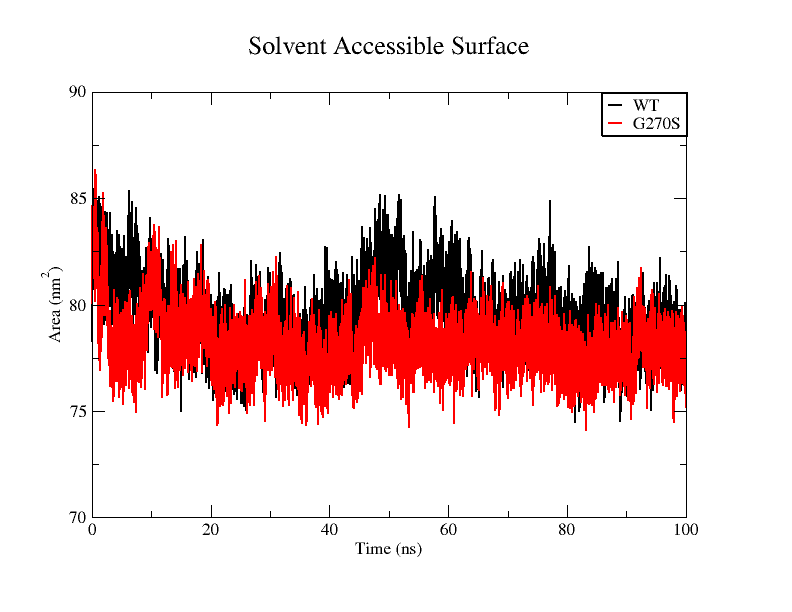 |  |
| **G270D** | **G270S** |  |

**Fig. S7** Visualization of secondary structure variations between wild-type and mutant CTRP6 C1q domain, marked with red rectangular box.


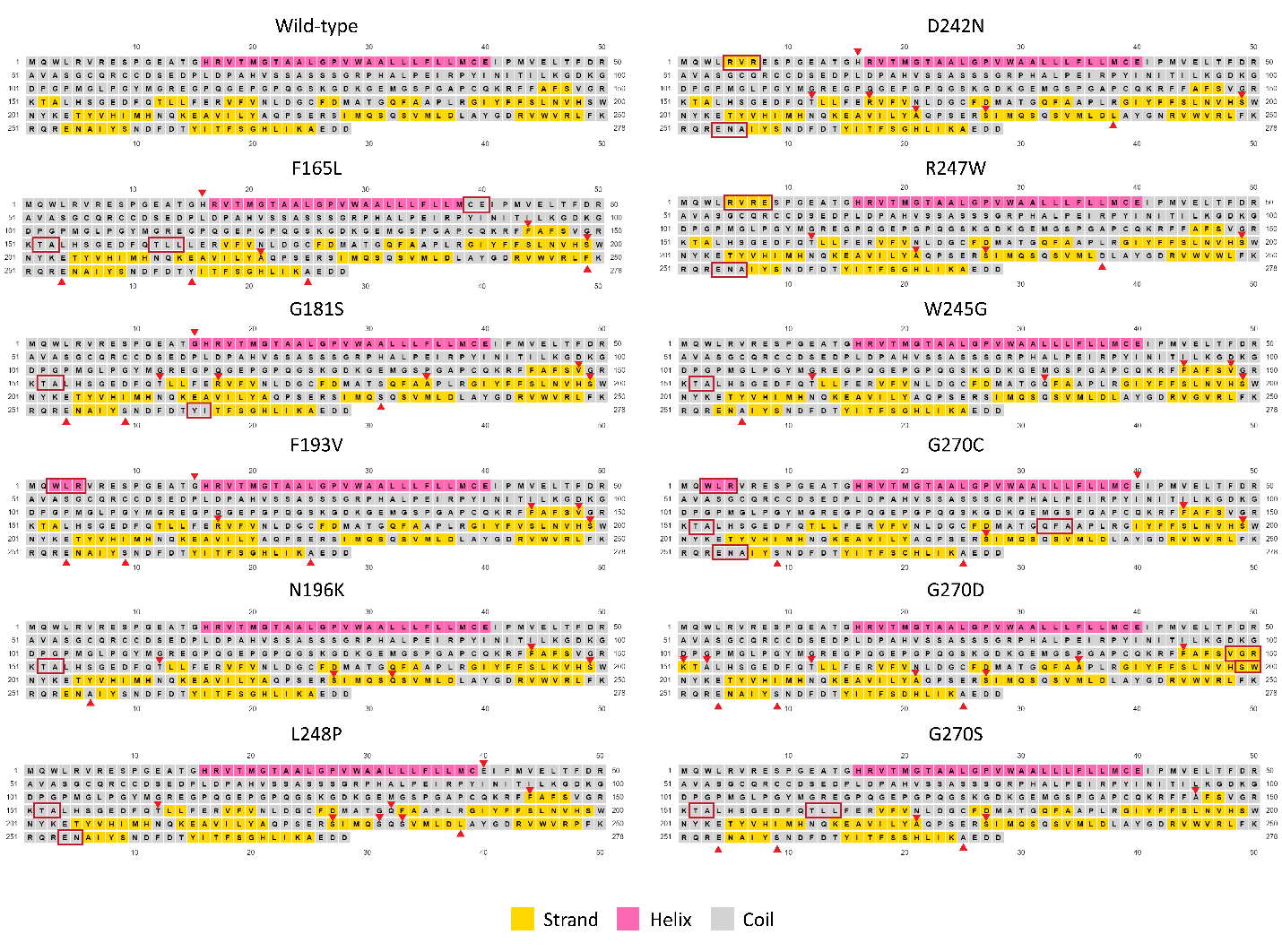

Supplement: Supplementary file 1 — Supplementary Material 1 [file 12885_2025_13685_MOESM1_ESM.docx]
